# Trends in health and health inequality during the Japanese economic stagnation: Implications for a healthy planet

**DOI:** 10.1016/j.ssmph.2023.101356

**Published:** 2023-02-06

**Authors:** Ayako Hiyoshi, Kaori Honjo, Loretta G. Platts, Yuka Suzuki, Martin J. Shipley, Hiroyasu Iso, Naoki Kondo, Eric J. Brunner

**Affiliations:** a Clinical Epidemiology and Biostatistics, School of Medical Sciences, Faculty of Medicine and Health, Örebro University, Örebro, Sweden; b Department of Epidemiology and Public Health, University College London, London, UK; c Department of Public Health Sciences, Stockholm University, Stockholm, Sweden; d Department of Social and Behavioural Sciences, Faculty of Medicine, Osaka Medical and Pharmaceutical University, Osaka, Japan; e Stress Research Institute, Department of Psychology, Stockholm University, Stockholm, Sweden; f Public Health, Department of Social Medicine, Osaka University Graduate School of Medicine, Osaka, Japan; g Institute for Global Health and Medicine, Bureau of International Health Cooperation, National Center for Global Health and Medicine, Tokyo, Japan; h Department of Social Epidemiology, Kyoto University, Kyoto, Japan

**Keywords:** Wellbeing, Self-rated health, Health inequalities, Planetary health, Sustainable development goals, GDP, Epidemiology, De-growth, Economic stagnation, GDP, Sustainable Development Goal, UK, The United Kingdom, US, The United States

## Abstract

**Introduction:**

Human health and wellbeing may depend on economic growth, the implication being that policymakers need to choose between population health and the health of ecosystems. Over two decades of low economic growth, Japan's life expectancy grew. Here we assess the temporal changes of subjective health and health inequality during the long-term low economic growth period.

**Methods:**

Eight triennial cross-sectional nationally representative surveys in Japan over the period of economic stagnation from 1992 to 2013 were used (n = 625,262). Health is defined positively as wellbeing, and negatively as poor health, based on self-rated health. We used Slope and Relative Indices of Inequality to model inequalities in self-rated health based on household income. Temporal changes in health and health inequalities over time were examined separately for children/adolescents, working-age adults, young-old and old-old.

**Results:**

At the end of the period of economic stagnation (2013), compared to the beginning (1992), the overall prevalence of wellbeing declined slightly in all age groups. However, poor health was stable or declined in the young-old and old-old, respectively, and increased only in working-age adults (Prevalence ratio: 1.14, 95% CI 1.08, 1.20, <0.001). Over time, inequality in wellbeing and poor self-rated health were observed in adults but less consistently for children, but the inequalities did not widen in any age group between the start and end of the stagnation period.

**Conclusions:**

Although this study was a case study of one country, Japan, and inference to other countries cannot be made with certainty, the findings provide evidence that low economic growth over two decades did not inevitably translate to unfavourable population health. Japanese health inequalities according to income were stable during the study period. Therefore, this study highlighted the possibility that for high-income countries, low economic growth may be compatible with good population health.

## Highlights

• Low economic growth may worsen mortality and life expectancy trends.
• We used a Japanese population-based survey series encompassing low growth period.
• A long period of low economic growth did not inevitably worsen population health.
• Low economic growth and population health are not a trade-off.

## 1 Introduction

Sustainable Development Goal (SDG) #8 refers to sustainable growth with no distinction between countries according to the state of economic development. There are compelling reasons to consider that high-income economics (Gross National Income per capita > $13,205) (The World Bank, 2021) should have less growth-focused development targets, with a transition towards a society managed well without economic growth, as suggested by advocates of economic degrowth (Kallis et al., 2018). Not least, resource consumption continues to run at unsustainable levels in Western Europe, the United States (US), and Japan despite more than three decades of warnings about the need to prevent further climate change and deterioration of the health of our planet by cutting the rate of global resource depletion (The Intergovernmental Panel on Climate Change) and destruction of Earth's natural systems.

In the context of developing mitigation measures and practices against climate change and environmental impacts, it is important to understand whether low economic growth is compatible with positive population health and health inequality trends (Ward et al., 2016; Whitmee et al., 2015). Historically, economic growth has been considered a positive factor for human progress and health (Keynes, 1936; Mckeown, 1976). However in recent decades it has become clear that measures of population health such as life expectancy and child mortality rates are only weakly associated with macroeconomic indices across high income countries (Preston, 1975). The present need to reduce resource and energy consumption thus prompts examination of the widespread orthodoxy that human wellbeing depends on continual economic growth. The implication for policy makers could be that it is not necessary to choose between measures to protect the planet and those that aim to improve population health.

A body of research has investigated the impact of economic growth and fluctuations, but the consequence of long-term economic stagnation among countries which achieved a high level of population health like Japan has not been investigated. Historically, in the long run, economic growth has been a positive factor for population health through nutrition improvements, enhancement in sanitation and housing, and advancements in medical technology (Frakt, 2018; Mckeown, 1976). On the other hand, findings about recent short-term economic downturns and population health have been mixed. Occurrence of mental health outcomes such as suicide often increase during economic downturns, while causes of death such as cancer may be less affected because of the long latency periods (Bezruchka, 2009; Ruhm, 2000). Deaths from causes such as traffic accidents, homicide as well as other chronic and non-chronic diseases may decline during economic downturns because of reduced industrial activity, reduced traffic accidents and air pollution, fewer working hours and less work stress. (Bezruchka, 2009; Dadgar & Norstrom, 2022; Granados, 2005; Ruhm, 2000). Further, the state and individuals may compensate economic loss by borrowing and utilising reserves. However, if economic contraction is prolonged, national and household resources may be depleted, with negative health consequences especially for economically disadvantaged groups. Japan entered a long period of low economic growth in the early 1990s (Okina et al., 2001), with a substantial drop in stock market values (Figure A1-A). During the subsequent two decades, the GDP growth rate for Japan was less than half of the UK and the US (Figure A1-B). The prosperity established in Japan by 1990, followed by long economic stagnation offers a valuable setting to examine the impact of a long-term low-performance economy on health.

Mortality-related health indicators are often used in the assessment of population health. Japan's population health indexed by all-cause mortality rate and life expectancy improved during the economic stagnation (OECD Data (a)). However, such overall trends may not reflect all aspects of health and even mask the deterioration of more subtle, less symptomatic pre-clinical phases of health. Easterlin suggested that economic growth predicts happiness in the short term but not in the long run because the growth of income in others vitiates the positive effects (Easterlin & O'connor, 2020). In a short-term recession, income and happiness decline together as seen during the 2007–2009 recessions in the US and Europe (Easterlin & O'connor, 2020), and both improve as economies recover. Self-rated health, a subjective measure of health, may be a useful measure because it has been shown to reflect physical and mental health including life satisfaction and wellbeing (Koots-Ausmees & Realo, 2015; La Parra-Casado et al., 2017; Roysamb et al., 2003) possibly because individuals incorporate consciously or unconsciously subtle preclinical and prodromal stages of health (Andresen et al., 2003; Idler & Benyamini, 1997).

The aim of this study is to examine the temporal changes in wellbeing and poor subjective health, and their corresponding health inequalities based on household income during the Japanese low economic growth period. We use a nationally representative survey series with almost two-thirds of a million participants surveyed in ten waves.

## 2 Materials and methods

### 2.1 Data

Ten triennial cross-sectional waves from 1986 to 2013 of nationally representative samples from the Comprehensive Survey of Living Conditions (CSLC) conducted by the Ministry of Health, Labour and Welfare were analysed. CSLC employs multi-stage stratified random cluster sampling with the primary sampling unit being census enumeration districts which divide Japan into approximately one million areas. After stratifying by prefectures and large cities, 5000 enumeration districts were randomly selected. All households and household members living in these areas were approached to complete the Demography and Health questionnaire. The response rate for this questionnaire was 96% in 1986 declining to 80% in 2013. A sub-sample residing in approximately 2000 randomly selected enumeration districts was further administered the Income and Saving questionnaire. Response rates for this subset are not available for the first three waves, thereafter ranging between 85% and 68%. Over the study period, no major changes occurred in the sampling procedure apart from the exclusion of one prefecture in 1995 due to an earthquake. We focus on data between 1992 and 2013, and results including the preceding period, 1986–1989, are included in the supplementary data. Of the entire sample, we used a subset that was administered all relevant questionnaires (n = 802,830) and aged 6–79 years. Participants with missing data on income after tax (n = 120,356) and self-rated health (n = 57,212) were excluded, resulting in the sample size of 632,177. Trends of health and health inequalities during the period of low economic growth may differ by age: working-age adults may be the most susceptible to changes in macro-economy due to subsequent labour market changes. Children may be influenced through their parents (Ueda et al., 2015). Constraints on health and social care expenditure and subsequent increases in out-of-pocket expenditure may affect the health of the older population. Therefore, we stratified the analysis by four age groups: children (6–19 years, n = 104,850), working-age adults (20–59 years, n = 381,852), young-old (60–69 years, n = 88,095), and old-old (70–79 years, n = 57,380).

### 2.2 Outcome variables: wellbeing and poor health

Health is analysed positively as wellbeing, and negatively as poor health based on self-rated health. These two conditions were analysed separately because they may not show identical changes over time. Self-rated health was assessed by a single question ‘What is your current health (condition)?’ with five categories of response: very good, good, normal, fair, and poor. The location of this question in the questionnaire was consistent across surveys. Wellbeing was defined as ‘very good’ or ‘good’ self-rated health, and poor health as ‘fair’ or ‘poor’ self-rated health. Self-rated health has been used in many surveys across continents (OECD, 2021) because, despite the brevity of the question, it is associated with physical and mental health (Andresen et al., 2003; Idler & Benyamini, 1997), with life satisfaction and wellbeing (Koots-Ausmees & Realo, 2015; La Parra-Casado et al., 2017; Roysamb et al., 2003) and further, is a good predictor of future health including mortality (DeSalvo et al., 2006; Lorem et al., 2020; Wuorela et al., 2020).

### 2.3 Main independent variables: survey year and income

Survey years was used as the independent variable. In the analysis of temporal trends, the year was used as a continuous variable centred at 1992. Annual household net income, including benefits and inheritance, *after* tax was equivalised to account for differences in household size by dividing it by the square root of the household size (OECD, 2020). Income deciles were created, stratified by survey year and age group with working-age adults internally stratified by younger (20–39 years) and older (40–59 years) adults to account for general income differences by age (Hiyoshi et al., 2013). Income *before* tax was also equivalised, made into deciles and used in a sensitivity analysis.

### 2.4 Other independent variables

5-year interval age, marital status (married, unmarried, widowed, and separated) and prefecture were treated as categorical variables. Marital status was used in analyses of adults but not children. We grouped Japan's 47 prefectures into nine regions to control for area differences in health and socioeconomic variables. A dichotomous employment status (yes or no) was used in a sensitivity analysis.

### 2.5 Statistical analysis

#### 2.5.1 Descriptive statistics

We calculated proportions and 95% confidence intervals for the proportions. Age- and gender-standardised prevalence proportions of wellbeing and poor health were calculated using direct standardization with the entire analytical sample as the standard population.

#### 2.5.2 Income inequalities over time

Large social inequalities tend to co-occur with large health inequalities. We calculated the Gini coefficient for household income before and after tax at each survey, adjusting for the mean age of adults ≥20 years in the household, the age and gender of the head of household, household size, and prefecture using the Wertz method, eliminating the inequality attributable to those variables.

#### 2.5.3 Time trend in prevalence proportion of wellbeing and poor health

Changes in prevalence were examined using two analyses: a direct comparison of 1992 and 2013, and an assessment of the trend over time, which uses all the data and reduces the effect of the volatility of the individual annual estimates. Using survey year as a continuous variable centred at 1992, probabilities of outcomes and 95% confidence intervals adjusting for age, gender, marital status and prefecture were estimated using binomial generalized linear models with a log link function based on cubic time trend, given the downward economy from 1992, a slight recovery in the early to the mid-2000s, and the Lehman shock in 2008 (Harari, 2013). All covariates were used as categorical variables.

#### 2.5.4 Inequalities in wellbeing and poor health

Prevalence of wellbeing and poor health were linearly related to income. Associations were summarised using Slope and Relative Indices of Inequality (SII and RII, respectively) using linear regression models for SII and binomial generalized linear models with a log link function for RII (Hiyoshi et al., 2013; Wagstaff et al., 1991). To calculate SII and RII, a cumulative rank variable was created by assigning values, ranging between 0 and 1, equal to the cumulative percentage of the midpoint of an income decile. For example, the lowest (tenth) income decile was assigned a score of approximately 0.95, and the ninth 0.85. The strength of the indices is their ability to provide a single summary measure of health disparity, including direction and magnitude, using data from all deciles. Estimates are interpreted as differences in predicted risk between the lowest and highest in the income hierarchy, taking into account the entire income distribution. SII is an absolute percentage difference, and RII is a risk ratio.

#### 2.5.5 Time trends in SII and RII

Changes in SII and RII over time were examined by comparing 1992 and 2013, and the time trends. First, to compare 1992 and 2013, models including an interaction term between the cumulative rank variable and a categorical year variable were fitted. Second, for time trends, a cubic time trend model was fitted by including three variables created by multiplying a cumulative rank variable with the calendar year (centred at 1992) with a power of 1, 2 or 3. If a cubic time trend variable was not statistically significant, a quadratic model was fitted. If a quadratic variable was not significant, a linear model was fitted. In all analyses, models were adjusted for all covariates.

Analyses were stratified by the four age groups because of the aforementioned reasons, and models for time trends of SII and RII differed by age group (likelihood ratio tests p < 0.05). The magnitude of SII and RII in 1992 tended to differ by gender, but inequality trends were parallel (likelihood ratio tests p > 0.05); therefore, gender was adjusted for. We present the gender-stratified estimates in the supplementary data.

All regression estimates were weighted using the sampling weights to account for non-response and sampling probability, and clustering of data by household was accounted for using a cluster sandwich estimator to give robust standard errors.

#### 2.5.6 Sensitivity analyses

Five sensitivity analyses were conducted. First, we used household income *before tax* instead of income *after tax*. Second, whether SII differed according to *employment status* was examined by including linear and quadratic time trend terms with their interaction with a binary employment status variable. Third, children aged 6–11 years were excluded because there is no clear evidence on small children's ratings of self-rated health, although they have been shown to rate the quality of life reliably (Breidablik et al., 2009; Varni et al., 2007). Fourth, we compared health and health inequalities in 2013 with 1986, the time of heightened economic performance of Japan. Fifth, we conducted multilevel multiple imputation for missing data for the survey years of 1992 and 2013 and re-examined change of prevalence, SII and RII between these two years. The imputation model included variables used in the analyses as well as household size, the presence or absence of a symptom needing treatment, a diagnosed disease and health conditions affecting daily life, type of employment and occupation if employed (otherwise included as no employment or children). Ten imputations were created, and imputed outcome was deleted before analysis (Von Hippel, 2007).

Analyses were conducted with Stata/SE15, and imputation was conducted using REALCOM.

## 3 Results

### 3.1 Gini coefficient

The adjusted Gini coefficient for income before tax remained broadly unchanged while that for income after tax tended to increase slightly (Figure A2, Appendix Table A1).

### 3.2 Time trends of wellbeing and poor health

Wellbeing declined by age, from 70% in children to 30% in old-old. Conversely, poor health increased from about 3% in children to over 20% in old-old (Table 1). Apart from poor health in children which was stable across the income deciles, wellbeing tended to increase and poor health to decrease by increasing income decile in all age groups. Age-standardized prevalence proportions are shown in the supplementary data Figure A3 and Table A2.

**Table 1:** Distribution of wellbeing and poor health according to the decile of income after tax and other characteristics, by age-group.

	Children			Working-age adults			Young-old			Old-old		
	(Ages 6–19)			(Ages 20–59)			(Ages 60–69)			(Ages 70–79)		
	(n = 104,850)			(n = 381,852)			(n = 88,095)			(n = 57,380)		
	Freq.	Well	Poor	Freq.	Well	Poor	Freq.	Well	Poor	Freq.	Well	Poor
		**(%)**	**(%)**		**(%)**	**(%)**		**(%)**	**(%)**		**(%)**	**(%)**
**Year**^a^												
1986				55,298	40	11	8428	31	19	4785	25	28
1989	22,968	66	3	61,551	46	10	11,169	33	18	6232	27	27
1992	19,543	71	2	55,925	51	9	11,391	37	16	6082	32	23
1995	15,544	70	2	48,263	52	8	10,572	39	15	5879	34	20
1998	12,226	64	3	41,323	46	9	9817	34	16	6072	31	24
2001	11,052	63	4	37,182	43	10	9669	34	17	7141	29	26
2004	6725	66	3	22,077	44	11	6500	35	17	5399	27	27
2007	6006	64	3	21,355	38	12	6116	29	19	5209	22	29
2010	5083	64	3	18,485	38	12	6523	29	16	4625	25	26
2013	5703	67	3	20,393	39	11	7910	31	14	5956	26	23
**Income**^b^												
Highest	10,529	69	3	38,301	49	8	9018	39	13	5888	31	21
2	10,626	68	3	38,475	47	9	8967	36	14	5816	28	24
3	10,548	68	3	38,490	46	9	8957	34	14	5768	28	23
4	10,565	68	3	38,325	45	9	8868	33	15	5817	28	25
5	10,470	66	3	38,334	45	9	8839	34	16	5773	29	23
6	10,445	65	3	38,261	44	10	8813	34	17	5746	27	26
7	10,439	66	3	38,212	44	10	8825	32	18	5680	29	26
8	10,404	65	3	38,114	44	10	8675	32	18	5643	28	27
9	10,434	65	3	37,803	43	12	8582	31	21	5564	27	28
Lowest	10,390	65	3	37,537	42	13	8551	30	23	5685	26	29
**Age**												
6–10	34,022	72	2									
11–14	31,486	68	3									
15–19	39,342	61	4									
20–24				39,736	56	6						
25–29				40,426	52	7						
30–34				44,649	49	8						
35–39				50,955	46	9						
40–44				52,629	45	9						
45–49				52,389	41	11						
50–54				51,639	39	13						
55–59				49,429	37	14						
60–64							47,386	35	15			
65–69							40,709	32	18			
70–74										32,792	30	23
75–79										24,588	26	28
**Gender**												
Male	53,472	67	3	185,087	47	9	41,234	36	16	24,619	31	24
Female	51,378	66	3	196,765	43	11	46,861	31	18	32,761	26	26
**Marital status**												
Married				276,332	44	10	71,624	34	16	37,506	29	25
Unmarried				87,607	51	8	2477	30	20	1077	27	25
Widowed				6343	36	15	11,063	32	18	17,452	27	25
Divorced				11,570	39	16	2931	29	23	1345	27	28

Freq: frequency, well: wellbeing, poor: poor health, %: row percentage.

a Self-rated health was not assessed for children in 1986.

b Income decile based on the household income after tax.

**Fig. 1:**
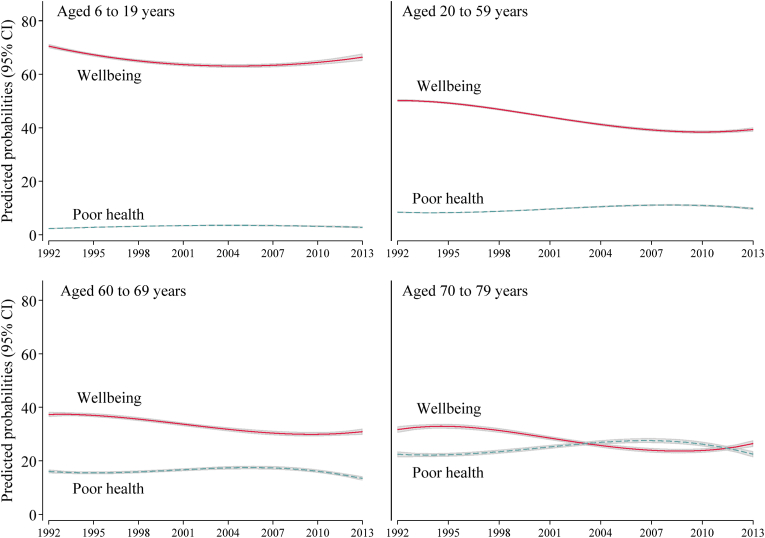
Predicted probabilities of age- and gender-adjusted wellbeing and poor self-rated health by age-group, 1992–2013. Binomial generalized linear model with log link function and quadratic time trend was used for children and cubic time trend terms were used for adults. Models were adjusted for 5-year interval age, gender, marital status and 9-group prefecture. For children, marital status was not included. For the calculation of probability, age and gender were held at mean levels. Coefficients are reported at supplementary data Tables A3.

There were small cubic time trends in adjusted wellbeing and poor health in all age groups except children (Table A3). In the adult age groups, there was initial stability in reporting wellbeing from 1992 to the mid-1990s, followed by a small decline until about 2005 (Fig. 1). When the prevalence in 2013 was compared with 1992, there were some declines in wellbeing in all age groups and increases in poor health in working-age adults over time in Model 2 (Table 2). In young-old, the rate of poor health declined, and in old-old, poor health did not change from 1992 to 2013. Adjustments for covariates did not change estimates notably.

**Table 2:** The ratio of the prevalence proportions of wellbeing and poor health in 2013 compared with 1992, by age-group.

Wellbeing	Model 1	P-value	Model 2	P-value
	Prevalence ratio		Prevalence ratio	
	(95% CI)		(95% CI)	
Children (6–19 years)	0.956 (0.931, 0.981)	0.001	0.952 (0.927, 0.978)	<0.001
Adults (20–59 years)	0.782 (0.764, 0.800)	<0.001	0.788 (0.769, 0.807)	<0.001
Young-old (60–69 years)	0.835 (0.797, 0.874)	<0.001	0.840 (0.802, 0.880)	<0.001
Old-old (70–79 years)	0.830 (0.780, 0.883)	<0.001	0.832 (0.781, 0.886)	<0.001

**Poor health**				
Children (6–19 years)	1.053 (0.868, 1.278)	0.601	1.075 (0.885, 1.306)	0.465
Adults (20–59 years)	1.186 (1.124, 1.251)	<0.001	1.136 (1.076, 1.200)	<0.001
Young-old (60–69 years)	0.865 (0.803, 0.931)	<0.001	0.824 (0.765, 0.888)	<0.001
Old-old (70–79 years)	1.015 (0.948, 1.088)	0.664	0.996 (0.928, 1.068)	0.910

Model 1: Adjusted for 5-year interval age and gender.

Model 2: Adjusted for 5-year interval age, gender, 9-group prefecture, and adult marital status.

All analyses were adjusted for data clustering by household and weighted.

### 3.3 Inequalities in wellbeing and the time trends

**Fig. 2:**
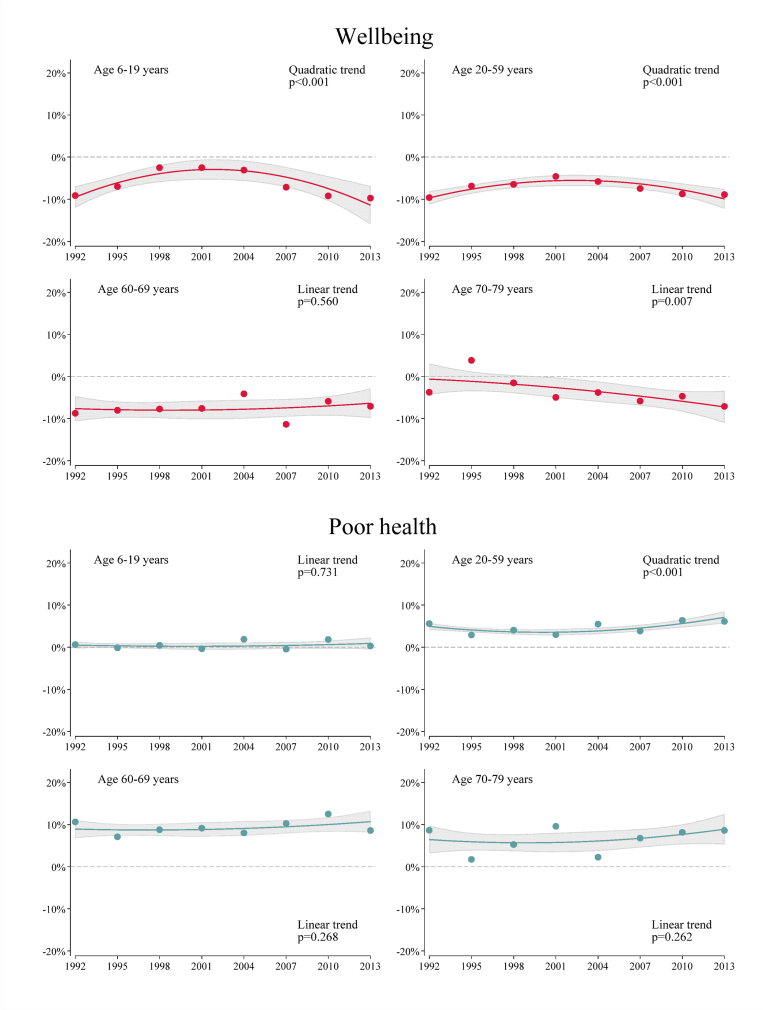
Slope Index of Inequality in wellbeing and poor health based on household income after tax by age-group, 1992–2013. Dots represent SII for each year calculated separately for each survey year (see: supplementary data Tables A4 for wellbeing and A6 for poor health). The information of SII for all years is provided at supplementary data Tables A4 and 6. Line and confidence intervals (shadowed area) were calculated using coefficients and standard errors obtained from a quadratic time trend model. Reported p-values are for a quadratic trend term if quadratic trend term was statistically significant. Otherwise, p-values for a linear trend term from a linear trend model which does not include a quadratic term in the model was reported. (see: supplementary data Tables A5 for wellbeing and A7 for poor health). All analyses were weighted using a survey weight and adjusted for categorical year, 5-year interval age, gender, marital status and 9-group prefecture, and robust standard errors were estimated using cluster sandwich estimator due to data clustering by household. In analysis of children, marital status was not included.

In 1992, among children, working-age adults and young-old, the report of wellbeing was approximately 10% lower in those with the lowest compared to highest income (Fig. 2, Table 3 and Table A4). There were no cubic trends in absolute inequality (SII). Inverse U-shaped quadratic trends in children and working-age adults and linear trends in young- and old-old were observed (Table A5). When health inequality in 2013 was compared with that at the beginning of the low growth period, in 1992, there was no statistically significant difference, indicating a similar magnitude of inequalities in 2013 and 1992 (Table 3). However, this finding may need to be interpreted with caution as there were linear increasing inequality trends in old-old, and SII in 1992 slightly deviated from the estimated trend (Fig. 2). Time trends in relative inequality (RII) were similar to those in absolute (Figure A4).

**Table 3:** Slope Index of Inequality (SII) for wellbeing and poor health for 1992 and 2013 and its change from 1992 to 2013, by age-group.

	Slope Index of Inequality^a^				Change in SII from 1992 to 2013^b^	
	1992	P-value	2013	P-value	Coef (95% CI)	P-value
	Coef (95% CI)		Coef (95% CI)			
**Wellbeing**						
Children (6–19 years)	−0.09 (−0.12, −0.06)	<0.001	−0.10 (−0.15, −0.04)	<0.001	0.0016 (−0.060, 0.063)	0.960
Adults (20–59 years)	−0.10 (−0.11, −0.08)	<0.001	−0.09 (−0.12, −0.06)	<0.001	0.0033 (−0.030, 0.036)	0.842
Young-old (60–69 years)	−0.09 (−0.12, −0.05)	<0.001	−0.07 (−0.11, −0.03)	0.001	0.022 (−0.031, 0.075)	0.413
Old-old (70–79 years)	−0.04 (−0.08, 0.01)	0.097	−0.07 (−0.12, −0.03)	0.002	−0.047 (−0.109, 0.015)	0.138
**Poor health**						
Children (6–19 years)	0.01 (−0.003, 0.02)	0.182	0.003 (−0.01, 0.02)	0.720	−0.0047 (−0.024, 0.014)	0.629
Adults (20–59 years)	0.06 (0.05, 0.07)	<0.001	0.06 (0.04, 0.08)	<0.001	0.0088 (−0.010, 0.028)	0.370
Young-old (60–69 years)	0.11 (0.08, 0.13)	<0.001	0.09 (0.05, 0.12)	<0.001	−0.015 (−0.054, 0.023)	0.439
Old-old (70–79 years)	0.09 (0.05, 0.13)	<0.001	0.09 (0.04, 0.13)	<0.001	0.00036 (−0.056, 0.057)	0.990

Coef: coefficient.

a Estimates were calculated by each survey year separately and adjusted for 5-year interval age, gender, marital status, and 9-group prefecture. All analyses were adjusted for data clustering by household and weighted. In analysis of children, marital status was not included.

b An interaction between the rank variable for SII and year (categorical, included only 1992 and 2013) is further included. The coefficient reported in the column is that for the interaction term, indicating the magnitude of change in SII for 2013 compared with 1992.

### 3.4 Inequalities in poor health and the time trends

Absolute inequality (SII) in poor health was around zero in children, 5% in working-age adults and 10% in young-old (Fig. 2, Table A6). The SII first narrowed and then widened in working-age adults (Table A7), returning to its initial level in 2013 (Table 3). In children, young-, and old-old, the SII was stable with no evidence of any trend. Time trends in relative inequality (RII) were similar to those in absolute, although confidence intervals were wide in children due to low prevalence (Figure A5).

### 3.5 Gender stratified analyses

Age-standardized prevalence of wellbeing and poor health showed largely parallel trends between men and women until the late 2000s when there was some convergence in both health outcomes in all age groups (Table A8 and Figure A6). In fully adjusted models, as the results combined both genders, wellbeing declined in all age groups in both men and women. However, poor health increased only in working-age adults, and there were some declines in young-old and remained unchanged in old-old (Table A9). The magnitude of inequalities, SIIs and RIIs, were similar between the two gender groups in children and working-age adults, but it was greater in men than in women of older ages, above 60 years. Time trends in SIIs and RIIs in both wellbeing and poor health were broadly similar between men and women and followed the similar pattern found in the results combined both genders (Tables A10-A15; Figures A7-A10).

### 3.6 Sensitivity analyses

First, analyses using income *after* tax produced similar SII and RII trends to those using income *before* tax (available on request). Second, there was no evidence of interaction between employment and SII time trends; therefore, the pattern of SII time trends was similar between those with and without employment (Table A16). Third, the exclusion of young children aged 6–11 years accentuated the reduction of wellbeing and increase of poor health in children from 1992 to 2013; it became evident that there was an approximately 40% increase in poor health (Table A17). The conclusion of stable inequalities in children, however, remained unchanged (Table A18). Fourth, when we compared 1986 (instead of 1992) to 2013, there was no decline in the prevalence of wellbeing, unlike the comparison between 1992 and 2013. However, the declines in poor health in older age groups, also now in working-age adults, as well as the absence of changes in SII over time broadly corresponded with the comparison between 1992 and 2013 (Table A19). Fifth, the comparison between 1992 and 2013 using the imputed data generated similar findings to that using unimputed data. Results showed that wellbeing declined in all ages, poor health increased in working-age but declined in older ages, and SII did not change (Tables A20-A21).

## 4 Discussion

This study set out to understand whether low economic growth is compatible with positive population health and health inequality trends in high-income countries using subjective health. The large-scale and representative data from Japan over two decades of low economic growth demonstrate that income inequality after tax based on the Gini coefficient worsened slightly, subjective wellbeing declined in general, and poor health in working-age increased to a degree during the long low economic growth period. However, poor health in older age sustained or improved and health inequality did not widen.

In our data, the fluctuation of prevalence proportions was greater in wellbeing than in poor health. Wellbeing in this study was defined by the most optimal two answers to the question of self-rated health, which is associated with happiness but may also be responsive to immediate increases in fear, worry, and stress in economic downturns (Deaton, 2012). It has been shown that suicide rates in the Japanese population are more sensitive to socioeconomic factors than in Western populations (Chen et al., 2009), and worries about life after retirement increased after 1992 (Ministry of Health, Labour and Welfare (MHLW, 2006). Thus, even if such concerns did not result in deterioration of health in older age, as shown in our study and also seen by national mortality and life expectancy trends (E-Stat: Portal Site for Official Statistics of Japan, 2022; OECD Data (b)), wellbeing, a measure that involves elements of emotional and psychological status, have fluctuated to a greater extent.

While we do not imply that subjective wellbeing could be sacrificed for the sake of the planet, the implication of our results is that the scope, or policy space, for introducing climate change mitigation measures and measures for a healthy planet is larger than the orthodoxy about the tight linkage of economic growth and a nation's health would suggest (Meurs et al., 2019). Health indicators closely influenced by emotional and psychological aspects may decline, but poor health as well as an objective measure of health – mortality and life expectancy – can continue to improve. Based on these, we consider that high-income countries can achieve and maintain good health without further economic growth, even sustainable growth, provided that policy measures to mitigate potential negative impacts on the emotional and psychological aspects of health are deployed. Some advocate sustainable degrowth, an emerging movement for a planned, as opposed to unintended, downscaling of production and consumption (Schneider et al., 2010; Van Den Bergh, 2011; Kallis et al., 2018). Pursuing SDGs, including the goal of population health, could be based on a highly differentiated strategy of investment and spending in low- and middle-income countries, and a less expansionary strategy in high-income countries. We can only speculate on the counterfactual situation of how health trends would have developed had the Japanese economy continued to expand at a higher rate after 1992. Annual GDP growth was less than half that in the US and UK (Japan 0.9%, US/UK >2.0%). Life expectancy at birth (LE_0_) in Japan was at that time the highest in the world after decades of strong economic growth (University of California, Berkeley (USA) & Max Planck Institute for demographic research (Germany)). LE_0_ increased by 3.2 years between 1992 and 2013, from 79.3 to 82.5 years, compared to increases of 4.6 years in the UK (76.3–80.9 years) and 4.2 years in the US (75.8–79.0 years) (University of California, Berkeley (USA) & Max Planck Institute for demographic research (Germany)). Men working in professional and managerial occupations experienced an increased mortality rate in the late 1990s (OECD Data (b)); thus, life expectancy in Japan might have been higher at the end of the study period if strong growth had continued. Nevertheless, Japan remained at the top of the international LE_0_ table in 2013.

Extrapolation of our findings to other high-income countries is very likely dependent on the broad policy context. Over the low growth period, economic inequality measured by household income after tax increased little (Figure A2). In 2015, the Gini coefficient for disposable income in Japan remained lower than that in UK and US, although higher than in Scandinavian countries. Furthermore, Japan's unemployment rate tended to remain lower than that of the US and UK (OECD Data (b)). Japan's per capita health expenditure was generally low, the second from the lowest among the Group of Seven (G7) countries in 1990, before the period of low economic growth (Ikeda et al., 2011; OECD Data (c)), and it remained lower relative to other G7 countries during the period of economic low growth. The continued policy emphasis on employment and material equality in Japan may contribute directly to the maintenance of national wellbeing. Indirectly, the Japanese perspective on wages and personal taxation is likely to reflect other health-promoting dimensions of collective social attitudes and policies, such as Japan's universal health care and public health system.

The CSLC survey is a nationally representative survey series with large sample size, allowing an opportunity to provide a good overview of subjective health and its socioeconomic inequalities over the entire Japanese economic stagnation period together with the 1986 and 1989 surveys in the last of the high growth years. Health and income information was collected similarly at all survey waves, enabling us to analyse trends of overall health, absolute and relative health inequality, and income inequality at the population level. The inclusion of children and older adults in the study population provided data on health during economic stagnation on the whole Japanese population except children aged ≤5, who were excluded due to the lack of data, and the elderly aged ≥80 years, who were excluded because it is assumed that sample was more selective due to survival, especially in the earlier survey years. We examined whether associations with wellbeing and poor health may differ by gender and employment and found broadly similar trends between groups defined by these variables.

A limitation of this study arises from the cross-sectional design of the CSLC series. Because each survey is based on a new sample we cannot examine the longitudinal relation between income and health in these data. For this reason, we present the trends in income inequality and health separately. The issue of causation is open to debate because, in principle, there may be a bidirectional relation between income and health and their inequalities. However, a large body of research in high-income countries shows the observed linkage in adults is that socioeconomic disadvantage leads to poor health (Elovainio et al., 2011; Kröger et al., 2015). Income was used as an indicator of socioeconomic circumstances including old age because income in older age, mostly pension, reflects lifetime earnings. This study was a case study of Japan focusing on the period of low economic growth using self-rated health and household income as the sole measure of health and social inequalities, respectively. The lack of comparisons limits the ability to derive conclusions and generalise the finding to other countries. In future research, the addition of other health and social measures and comparisons, including periods of prosperity, and data for other populations, would provide further insight.

## 5 Conclusions

In this case study of Japan, although subjective wellbeing deteriorated in general, poor health in older adults and health inequalities, assessed by 10 triennial repeated questionnaires in nationally representative surveys, did not exacerbate in 2013, at the end of Japan's low growth period, compared with 1992, the beginning of the long stagnation period. We cannot extrapolate results to other settings, but this study has highlighted possibilities that in high-income countries, high GDP growth may not be a prerequisite for positive population health and health inequalities, and long-term low economic growth can be compatible with stable and improving population health.

## Ethics statement

Ethics approval was not necessary because the data were collected by the Ministry of Health, Labour and Welfare (MHLW) under the Statistics Act, and our data provided by MHLW did not contain any individual identifiable information.

## Declaration of competing interest

The authors declare that they have no known competing financial interests or personal relationships that could have appeared to influence the work reported in this paper.

## Data Availability

The authors do not have permission to share data.
