# Supplementary material for: Trends in health and health inequality during the Japanese economic stagnation: Implications for a healthy planet
Source: SSM Popul Health. 2023 Feb 6;22:101356. doi: 10.1016/j.ssmph.2023.101356 (PMC9958394; doi:10.1016/j.ssmph.2023.101356)
Supplement: Multimedia component 1 [file mmc1.docx]

**SUPPLEMENTARY DATA**

Supplement to: (Author names). **Trends in health and health inequality during the Japanese economic stagnation: implications for a healthy planet**

**Contents**

[TEXT 3](#_Toc93943406)

[A1 Description of the Comprehensive Survey of Living Conditions (CSLC) survey 3](#_Toc93943407)

[TABLES 4](#_Toc93943408)

[TABLE A1 Adjusted Gini coefficients for household income before and after tax, 1986-2013 4](#_Toc93943409)

[TABLE A2 Distribution of wellbeing and poor health according to the decile of income after tax and other characteristics, by age-group 5](#_Toc93943410)

[TABLE A3 Age- and gender-standardized prevalence rates and 95% confidence intervals for wellbeing and poor health, 1986-2013 6](#_Toc93943411)

[TABLE A4 Time trend of wellbeing and poor health by age-group, 1992-2013 7](#_Toc93943412)

[TABLE A5 Slope and Relative Indices of Inequality in wellbeing based on household income after tax by age-group, 1986-2013 8](#_Toc93943413)

[TABLE A6 Time trends in the Slope and Relative Indices of Inequality in wellbeing based on household income after tax by age-group, 1992-2013 9](#_Toc93943414)

[TABLE A7 Slope and Relative Indices of Inequality in poor health based on household income after tax by age-group, 1986-2013 10](#_Toc93943415)

[TABLE A8 Time trends for the Slope and Relative Indices of Inequality in poor health based on household income after tax by age-group, 1992-2013 11](#_Toc93943416)

[TABLE A9 Age-standardized prevalence rates and 95% confidence intervals for wellbeing and poor health by age-group and gender, 1986-2013 12](#_Toc93943417)

[TABLE A10 The ratio of the prevalence proportions of wellbeing and poor health in 2013 compared with 1992, by age-group and gender 13](#_Toc93943418)

[TABLE A11 Slope and Relative Indices of Inequality in wellbeing based on household income after tax by age-group and gender, 1986-2013 14](#_Toc93943419)

[TABLE A12 Time trends for the Slope and Relative Indices of Inequality in wellbeing based on household income after tax by age-group and gender, 1992-2013 15](#_Toc93943420)

[TABLE A13 The comparison of Slope and Relative Indices of Inequality in wellbeing based on household income after tax in 1992 and 2013, by age-group and gender 16](#_Toc93943421)

[TABLE A14 Slope and Relative Indices of Inequality in poor health based on household income after tax by age-group and gender, 1986-2013 17](#_Toc93943422)

[TABLE A15 Time trends for the Slope and Relative Indices of Inequality in poor health based on household income after tax by age-group and gender, 1992-2013 18](#_Toc93943423)

[TABLE A16 The comparison of Slope and Relative Indices of Inequality in poor health based on household income after tax in 1992 and 2013, by age-group and gender 19](#_Toc93943424)

[TABLE A17 Interaction of the Slope Index of Inequality in wellbeing and poor health based on income after tax with employment status, 1992-2013 20](#_Toc93943425)

[TABLE A18 The ratio of the prevalence proportions of wellbeing and poor health in 2013 compared with 1992 for children excluding aged 6-11 years 21](#_Toc93943426)

[TABLE A19 Slope Index of Inequality for wellbeing and poor health in 2013 compared with 1992 for children excluding aged 6-11 years 22](#_Toc93943427)

[TABLE A20 The ratio of the prevalence proportion of wellbeing and poor health in 2013 compared with 1986, and the Slope and Relative Indices of Inequality in wellbeing and poor health in 2013 compared with 1986, by age-group 23](#_Toc93943428)

[TABLE A21 The ratio of the prevalence proportions of wellbeing and poor health in 2013 compared with 1992, by age-group, using sample after multilevel multiple imputation 24](#_Toc93943429)

[TABLE A22 Slope Index of Inequality for wellbeing and poor health in 2013 compared with 1992, by age-group, using sample after multilevel multiple imputation 25](#_Toc93943430)

[FIGURES 26](#_Toc93943431)

[FIGURE A1 (A) Historical trends in the highest Nikkei Stock Average values on 1 January for Japan, and (B) GDP growth rates for Japan, United Kingdom and United States 26](#_Toc93943432)

[FIGURE A2 Inequality (adjusted Gini coefficient) in household income before and after tax, 1986-2013 27](#_Toc93943433)

[FIGURE A3 Prevalence of age- and gender-standardized wellbeing and poor health by age-group, 1986-2013 28](#_Toc93943434)

[FIGURE A4 Relative Index of Inequality in wellbeing based on household income after tax by age-group, 1992-2013 29](#_Toc93943435)

[FIGURE A5 Relative Index of Inequality in poor health based on household income after tax for age-group, 1992-2013 30](#_Toc93943436)

[FIGURE A6 Prevalence of age-standardized wellbeing and poor health by age-group and gender, 1986-2013 31](#_Toc93943437)

[FIGURE A7 Slope Index of Inequality in wellbeing based on household income after tax for age-group and gender, 1992-2013 32](#_Toc93943438)

[FIGURE A8 Slope Index of Inequality in poor health based on household income after tax for age-group and gender, 1992-2013 33](#_Toc93943439)

[FIGURE A9 Relative Index of Inequality in wellbeing based on household income after tax for age-group and gender, 1992-2013 34](#_Toc93943440)

[FIGURE A10 Relative Index of Inequality in poor health based on household income after tax for age-group and gender, 1992-2013 35](#_Toc93943441)

# TEXT

## A1 Description of the Comprehensive Survey of Living Conditions (CSLC) survey

CSLC employs multi-stage stratified random cluster sampling with the primary sampling unit being census enumeration districts (EDs) which divide Japan into approximately one million areas. After stratifying by prefectures and large cities, 5000 EDs were randomly selected. All households and household members living in these areas were approached to complete the Demography & Health questionnaire. The response rate was 96% in 1986 declining to 80% in 2013. A sub-sample residing in approximately 2000 randomly selected EDs was administered the Income & Saving questionnaire. Response rates for this subset are not available for the first three waves, thereafter ranging between 85% and 68%. Over the study period, no major changes occurred in the sampling procedure apart from exclusion of one prefecture in 1995 due to an earthquake.

Back to the contents list (p.1)

# TABLES

## TABLE A1 Adjusted Gini coefficients for household income before and after tax, 1986-2013

| **Household income** | **Before tax** | **After tax** |
| --- | --- | --- |
|  |  |  |
| 1986 | 0.314 | 0.290 |
| 1989 | 0.323 | 0.307 |
| 1992 | 0.325 | 0.305 |
| 1995 | 0.329 | 0.308 |
| 1998 | 0.330 | 0.314 |
| 2001 | 0.343 | 0.332 |
| 2004 | 0.316 | 0.313 |
| 2007 | 0.330 | 0.318 |
| 2010 | 0.331 | 0.323 |
| 2013 | 0.328 | 0.324 |
|  |  |  |

Back to the contents list (p.1)


## TABLE A3 Age- and gender-standardized prevalence rates and 95% confidence intervals for wellbeing and poor health, 1986-2013

| **Year** | | **Wellbeing**  **(95% CI)** | **Poor health**  **(95% CI)** |
| --- | --- | --- | --- |
|  |  | | |
| **Children (6 to 19 years)** | | | |
|  | | | |
| 1989 | | 66 (65, 67) | 3 (3, 3) |
| 1992 | | 71 (70, 71) | 2 (2, 3) |
| 1995 | | 70 (69, 71) | 3 (2, 3) |
| 1998 | | 64 (63, 65) | 4 (3, 4) |
| 2001 | | 63 (62, 63) | 4 (3, 4) |
| 2004 | | 65 (64, 67) | 3 (3, 4) |
| 2007 | | 63 (62, 65) | 4 (3, 4) |
| 2010 | | 63 (62, 64) | 4 (3, 4) |
| 2013 | | 66 (65, 68) | 3 (2, 3) |
|  | |  |  |
| **Adults (20 to 59 years)** | | | |
|  |  | | |
| 1986 | | 40 (40, 41) | 11 (10, 11) |
| 1989 | | 46 (45, 46) | 10 (10, 10) |
| 1992 | | 50 (50, 51) | 9 (9, 9) |
| 1995 | | 52 (51, 52) | 8 (8, 8) |
| 1998 | | 46 (46, 47) | 10 (9, 10) |
| 2001 | | 44 (43, 44) | 10 (10, 10) |
| 2004 | | 45 (44, 45) | 11 (10, 11) |
| 2007 | | 39 (38, 40) | 11 (11, 12) |
| 2010 | | 38 (37, 39) | 12 (11, 12) |
| 2013 | | 39 (39, 40) | 10 (10, 11) |
|  | |  |  |
| **Young-old (60 to 69 years)** | | | |
|  |  | | |
| 1986 | | 31 (30, 32) | 19 (19, 20) |
| 1989 | | 33 (32, 34) | 19 (18, 19) |
| 1992 | | 37 (36, 38) | 16 (16, 17) |
| 1995 | | 39 (38, 40) | 15 (14, 15) |
| 1998 | | 34 (33, 35) | 16 (15, 17) |
| 2001 | | 34 (33, 35) | 17 (16, 18) |
| 2004 | | 35 (34, 36) | 17 (16, 18) |
| 2007 | | 29 (28, 30) | 19 (18, 20) |
| 2010 | | 29 (28, 30) | 17 (16, 17) |
| 2013 | | 31 (30, 32) | 14 (13, 15) |
|  | |  |  |
| **Old-old (70 to 79 years)** | | | |
|  |  | | |
| 1986 | | 25 (24, 27) | 28 (27, 29) |
| 1989 | | 27 (26, 28) | 27 (26, 28) |
| 1992 | | 32 (31, 33) | 23 (22, 24) |
| 1995 | | 34 (33, 35) | 21 (19, 22) |
| 1998 | | 31 (29, 32) | 24 (23, 25) |
| 2001 | | 29 (28, 30) | 26 (25, 27) |
| 2004 | | 27 (26, 29) | 27 (26, 28) |
| 2007 | | 22 (21, 23) | 28 (27, 30) |
| 2010 | | 25 (23, 26) | 25 (24, 27) |
| 2013 | | 26 (25, 27) | 23 (22, 24) |
|  | |  |  |

Directly standardized. The entire analysis sample was used as reference population.

Back to the contents list (p.1)

## TABLE A4 Time trend of wellbeing and poor health by age-group, 1992-2013

|  | **Wellbeing** | **Poor health** |
| --- | --- | --- |
|  |  |  |
|  | **Risk ratio (95% CI), p-value** | **Risk ratio (95% CI), p-value** |
|  |  |  |
| **Children (6 to 19 years)** |  |  |
|  |  |  |
| Linear trend term | 0.982 (0.979, 0.985), <0.001 | 1.073 (1.050, 1.096), <0.001 |
| Quadratic trend term | 1.001 (1.001, 1.001), <0.001 | 0.997 (0.996, 0.998), <0.001 |
| Cubic trend term | Not applicable | Not applicable |
|  |  |  |
| **Adults (20 to 59 years)** |  |  |
|  |  |  |
| Linear trend term | 1.000 (0.995, 1.006), 0.872 | 0.982 (0.968, 0.995), 0.009 |
| Quadratic trend term | 0.997 (0.997, 0.998), <0.001 | 1.006 (1.004, 1.007), <0.001 |
| Cubic trend term | 1.0001 (1.0001, 1.0001), <0.001 | 0.9998 (0.9997, 0.9998), <0.001 |
|  |  |  |
| **Young-old (60 to 69 years)** |  |  |
|  |  |  |
| Linear trend term | 1.005 (0.992, 1.017), 0.473 | 0.975 (0.955, 0.995), 0.015 |
| Quadratic trend term | 0.997 (0.996, 0.999), 0.001 | 1.005 (1.003, 1.008), <0.001 |
| Cubic trend term | 1.0001 (1.00004, 1.0001), <0.001 | 0.9998 (0.9997, 0.9999), <0.001 |
|  |  |  |
| **Old-old (70 to 79 years)** |  |  |
|  |  |  |
| Linear trend term | 1.031 (1.013, 1.049), 0.001 | 0.985 (0.965, 1.005), 0.147 |
| Quadratic trend term | 0.993 (0.991, 0.995), <0.001 | 1.005 (1.003, 1.007), <0.001 |
| Cubic trend term | 1.0002 (1.0002, 1.0003), <0.001 | 0.9998 (0.9997, 0.9999), <0.001 |
|  |  |  |

Estimates were obtained from a cubic time trend model using binomial generalized linear models. For children, estimates were obtained from a quadratic time trend model. Regression models were adjusted for 5-year interval age, gender and 9-group prefecture. In the analysis of children, marital status was not included. All analyses were adjusted for data clustering by household and weighted.

Back to the contents list (p.1)

## TABLE A4 Slope and Relative Indices of Inequality in wellbeing based on household income after tax by age-group, 1986-2013

| **Year** | **Slope Index of Inequality**  **(95% CI)** | **Relative Index of Inequality**  **(95% CI)** |
| --- | --- | --- |
|  |  |  |
| **Children (6 to 19 years)** | | |
|  |  |  |
| 1989 | -0.08 (-0.10, -0.05) | 0.89 (0.85, 0.93) |
| 1992 | -0.09 (-0.12, -0.06) | 0.87 (0.84, 0.91) |
| 1995 | -0.07 (-0.10, -0.04) | 0.90 (0.86, 0.94) |
| 1998 | -0.03 (-0.06, 0.01) | 0.96 (0.90, 1.01) |
| 2001 | -0.03 (-0.07, 0.02) | 0.96 (0.89, 1.03) |
| 2004 | -0.03 (-0.08, 0.02) | 0.93 (0.86, 1.01) |
| 2007 | -0.07 (-0.13, -0.02) | 0.89 (0.81, 0.98) |
| 2010 | -0.09 (-0.15, -0.03) | 0.87 (0.79, 0.96) |
| 2013 | -0.10 (-0.15, -0.04) | 0.89 (0.82, 0.96) |
|  |  |  |
| **Adults (20 to 59 years)** | | |
|  |  | |
| 1986 | -0.06 (-0.08, -0.04) | 0.86 (0.82, 0.90) |
| 1989 | -0.06 (-0.07, -0.04) | 0.89 (0.86, 0.93) |
| 1992 | -0.10 (-0.11, -0.08) | 0.83 (0.80, 0.86) |
| 1995 | -0.07 (-0.09, -0.05) | 0.88 (0.85, 0.92) |
| 1998 | -0.06 (-0.09, -0.04) | 0.88 (0.84, 0.92) |
| 2001 | -0.05 (-0.07, -0.02) | 0.90 (0.85, 0.95) |
| 2004 | -0.06 (-0.09, -0.03) | 0.88 (0.82, 0.94) |
| 2007 | -0.07 (-0.10, -0.05) | 0.83 (0.77, 0.89) |
| 2010 | -0.09 (-0.12, -0.06) | 0.79 (0.73, 0.86) |
| 2013 | -0.09 (-0.12, -0.06) | 0.79 (0.74, 0.85) |
|  |  |  |
| **Young-old (60 to 69 years)** | | |
|  |  | |
| 1986 | -0.06 (-0.10, -0.03) | 0.81 (0.72, 0.92) |
| 1989 | -0.06 (-0.09, -0.03) | 0.84 (0.75, 0.93) |
| 1992 | -0.09 (-0.12, -0.05) | 0.79 (0.72, 0.87) |
| 1995 | -0.08 (-0.12, -0.04) | 0.81 (0.74, 0.89) |
| 1998 | -0.08 (-0.11, -0.04) | 0.80 (0.72, 0.89) |
| 2001 | -0.08 (-0.12, -0.04) | 0.79 (0.70, 0.89) |
| 2004 | -0.04 (-0.09, 0.01) | 0.89 (0.77, 1.02) |
| 2007 | -0.11 (-0.16, -0.07) | 0.67 (0.57, 0.79) |
| 2010 | -0.06 (-0.11, -0.01) | 0.83 (0.70, 0.97) |
| 2013 | -0.07 (-0.11, -0.03) | 0.80 (0.69, 0.91) |
|  |  |  |
| **Old-old (70 to 79 years)** | | |
|  |  | |
| 1986 | 0.02 (-0.02, 0.07) | 1.09 (0.91, 1.30) |
| 1989 | -0.05 (-0.09, -0.01) | 0.83 (0.71, 0.97) |
| 1992 | -0.04 (-0.08, 0.01) | 0.88 (0.76, 1.01) |
| 1995 | 0.04 (-0.01, 0.08) | 1.12 (0.97, 1.28) |
| 1998 | -0.02 (-0.06, 0.03) | 0.95 (0.82, 1.10) |
| 2001 | -0.05 (-0.09, -0.01) | 0.84 (0.72, 0.97) |
| 2004 | -0.04 (-0.09, 0.01) | 0.86 (0.72, 1.04) |
| 2007 | -0.06 (-0.10, -0.01) | 0.76 (0.62, 0.94) |
| 2010 | -0.05 (-0.10, 0.00) | 0.81 (0.66, 1.00) |
| 2013 | -0.07 (-0.12, -0.03) | 0.76 (0.64, 0.91) |
|  |  |  |

Estimates were calculated by each survey year separately and adjusted for 5-year interval age, gender, marital status, and 9-group prefecture. All analyses were adjusted for data clustering by household and weighted. In analysis of children, marital status was not included.

Back to the contents list (p.1)

## TABLE A6 Time trends in the Slope and Relative Indices of Inequality in wellbeing based on household income after tax by age-group, 1992-2013

|  |  | **Slope Index of Inequality** |  | **Relative Index of Inequality** |
| --- | --- | --- | --- | --- |
|  |  |  |  |  |
|  |  | **Coefficient (95% CI), p-value** |  | **Coefficient (95% CI), p-value** |
| **Children (6 to 19 years)** |  |  |  |  |
|  |  |  |  |  |
| Main effect (in 1992) |  | -0.095 (-0.120, -0.070), <0.001 |  | 0.868 (0.836, 0.900), <0.001 |
| Linear trend term |  | 0.013 (0.0062, 0.020), <0.001 |  | 1.018 (1.007, 1.029), 0.001 |
| Quadratic trend term |  | -0.001 (-0.0010, 0.00034), <0.001 |  | 0.999 (0.999, 1.000), 0.001 |
|  |  |  |  |  |
| **Adults (20 to 59 years)** |  |  |  |  |
|  |  |  |  |  |
| Main effect (in 1992) |  | -0.097 (-0.113, -0.081), <0.001 |  | 0.823 (0.797, 0.850), <0.001 |
| Linear trend term |  | 0.0080 (0.0040, 0.012), <0.001 |  | 1.017 (1.008, 1.026), <0.001 |
| Quadratic trend term |  | 0.00039 (-0.00059, 0.00019), <0.001 |  | 0.999 (0.999, 1.000), <0.001 |
|  |  |  |  |  |
| **Young-old (60 to 69 years)** |  |  |  |  |
|  |  |  |  |  |
| Main effect (in 1992) |  | -0.081 (-0.104, -0.058), <0.001 |  | 0.808 (0.758, 0.861), <0.001 |
| Linear trend term |  | 0.00059 (-0.0014, 0.0026), 0.560 |  | 0.999 (0.993, 1.005), 0.699 |
| Quadratic trend term |  | Not applicable |  | Not applicable |
|  |  |  |  |  |
| **Old-old (70 to 79 years)** |  |  |  |  |
|  |  |  |  |  |
| Main effect (in 1992) |  | -0.0012 (-0.030, 0.027), 0.935 |  | 1.004 (0.916, 1.099), 0.939 |
| Linear trend term |  | -0.0032 (-0.0054, -0.00088), 0.007 |  | 0.987 (0.979, 0.995), 0.001 |
| Quadratic trend term |  | Not applicable |  | Not applicable |
|  |  |  |  |  |

For children and adults, estimates were obtained from a quadratic time trend model. For the young- and old-old groups, estimates were obtained from a linear time trend model.

Each model was adjusted for categorical year, 5-year interval age, gender, marital status and 9-group prefecture. In analysis of children, marital status was not included. All analyses were adjusted for data clustering by household and weighted.

Back to the contents list (p.1)

## TABLE A6 Slope and Relative Indices of Inequality in poor health based on household income after tax by age-group, 1986-2013

| **Year** | **Slope Index of Inequality**  **(95% CI)** | **Relative Index of Inequality**  **(95% CI)** |
| --- | --- | --- |
|  |  |  |
| **Children (6 to 19 years)** | | |
|  |  |  |
| 1989 | 0.01 (0.00, 0.01) | 1.22 (0.92, 1.62) |
| 1992 | 0.01 (0.00, 0.02) | 1.26 (0.90, 1.76) |
| 1995 | 0.00 (-0.01, 0.01) | 0.95 (0.68, 1.32) |
| 1998 | 0.00 (-0.01, 0.02) | 1.13 (0.80, 1.60) |
| 2001 | 0.00 (-0.02, 0.01) | 0.91 (0.60, 1.37) |
| 2004 | 0.02 (0.00, 0.04) | 1.64 (0.98, 2.75) |
| 2007 | 0.00 (-0.02, 0.01) | 0.87 (0.53, 1.42) |
| 2010 | 0.02 (0.00, 0.04) | 1.65 (0.96, 2.84) |
| 2013 | 0.00 (-0.01, 0.02) | 1.12 (0.63, 2.01) |
|  |  |  |
| **Adults (20 to 59 years)** | | |
|  |  | |
| 1986 | 0.04 (0.03, 0.05) | 1.49 (1.35, 1.64) |
| 1989 | 0.04 (0.03, 0.05) | 1.53 (1.39, 1.67) |
| 1992 | 0.06 (0.05, 0.07) | 1.90 (1.71, 2.12) |
| 1995 | 0.03 (0.02, 0.04) | 1.45 (1.28, 1.64) |
| 1998 | 0.04 (0.03, 0.05) | 1.53 (1.36, 1.72) |
| 2001 | 0.03 (0.02, 0.04) | 1.33 (1.17, 1.51) |
| 2004 | 0.06 (0.04, 0.07) | 1.64 (1.41, 1.92) |
| 2007 | 0.04 (0.02, 0.06) | 1.37 (1.18, 1.59) |
| 2010 | 0.06 (0.04, 0.08) | 1.70 (1.44, 1.99) |
| 2013 | 0.06 (0.04, 0.08) | 1.78 (1.51, 2.09) |
|  |  |  |
| **Young-old (60 to 69 years)** | | |
|  |  | |
| 1986 | 0.08 (0.05, 0.11) | 1.53 (1.29, 1.81) |
| 1989 | 0.09 (0.06, 0.11) | 1.61 (1.38, 1.88) |
| 1992 | 0.11 (0.08, 0.13) | 1.94 (1.65, 2.27) |
| 1995 | 0.07 (0.05, 0.10) | 1.64 (1.37, 1.95) |
| 1998 | 0.09 (0.06, 0.12) | 1.74 (1.45, 2.07) |
| 2001 | 0.09 (0.06, 0.12) | 1.71 (1.43, 2.04) |
| 2004 | 0.08 (0.04, 0.12) | 1.61 (1.30, 1.99) |
| 2007 | 0.10 (0.06, 0.14) | 1.77 (1.42, 2.21) |
| 2010 | 0.13 (0.09, 0.16) | 2.21 (1.77, 2.77) |
| 2013 | 0.09 (0.05, 0.12) | 1.88 (1.50, 2.34) |
|  |  |  |
| **Old-old (70 to 79 years)** | | |
|  |  | |
| 1986 | 0.05 (0.00, 0.10) | 1.20 (1.01, 1.43) |
| 1989 | 0.10 (0.06, 0.14) | 1.42 (1.22, 1.66) |
| 1992 | 0.09 (0.05, 0.13) | 1.44 (1.21, 1.71) |
| 1995 | 0.02 (-0.02, 0.06) | 1.09 (0.90, 1.31) |
| 1998 | 0.05 (0.01, 0.09) | 1.24 (1.05, 1.47) |
| 2001 | 0.10 (0.05, 0.14) | 1.43 (1.22, 1.68) |
| 2004 | 0.02 (-0.02, 0.07) | 1.07 (0.90, 1.28) |
| 2007 | 0.07 (0.02, 0.12) | 1.25 (1.05, 1.49) |
| 2010 | 0.08 (0.03, 0.13) | 1.36 (1.12, 1.66) |
| 2013 | 0.09 (0.04, 0.13) | 1.44 (1.20, 1.74) |
|  |  |  |

Estimates were calculated by each survey year separately and adjusted for 5-year interval age, gender, marital status, 9-group prefecture. All analyses were adjusted for data clustering by household and weighted. In analysis of children, marital status was not included.

Back to the contents list (p.1)

## TABLE A8 Time trends for the Slope and Relative Indices of Inequality in poor health based on household income after tax by age-group, 1992-2013

|  |  | **Slope Index of Inequality** |  | **Relative Index of Inequality** |
| --- | --- | --- | --- | --- |
|  |  |  |  |  |
|  |  | **Coefficient (95% CI), p-value** |  | **Coefficient (95% CI), p-value** |
| **Children (6 to 19 years)** |  |  |  |  |
|  |  |  |  |  |
| Main effect (in 1992) |  | 0.0032 (-0.0034, 0.0097), 0.342 |  | 1.097 (0.874, 1.376), 0.425 |
| Linear trend term |  | 0.00012 (-0.00056, 0.00080), 0.731 |  | 1.004 (0.982, 1.027), 0.708 |
| Quadratic trend term |  | Not applicable |  | Not applicable |
|  |  |  |  |  |
| **Adults (20 to 59 years)** |  |  |  |  |
|  |  |  |  |  |
| Main effect (in 1992) |  | 0.049 (0.041, 0.057), <0.001 |  | 1.808 (1.650, 1.981), <0.001 |
| Linear trend term |  | -0.0034 (-0.0057, -0.0012), 0.003 |  | 0.952 (0.930, 0.974), <0.001 |
| Quadratic trend term |  | 0.00021 (0.00010, 0.00032), <0.001 |  | 1.002 (1.001, 1.004), <0.001 |
|  |  |  |  |  |
| **Young-old (60 to 69 years)** |  |  |  |  |
|  |  |  |  |  |
| Main effect (in 1992) |  | 0.085 (0.068, 0.101), <0.001 |  | 1.685 (1.512, 1.879), <0.001 |
| Linear trend term |  | 0.00083 (-0.00064, 0.0023), 0.268 |  | 1.006 (0.997, 1.016), 0.196 |
| Quadratic trend term |  | Not applicable |  | Not applicable |
|  |  |  |  |  |
| **Old-old (70 to 79 years)** |  |  |  |  |
|  |  |  |  |  |
| Main effect (in 1992) |  | 0.054 (0.028, 0.079), <0.001 |  | 1.254 (1.122, 1.400), <0.001 |
| Linear trend term |  | 0.0012 (-0.00091, 0.0033), 0.262 |  | 1.003 (0.994, 1.012), 0.475 |
| Quadratic trend term |  | Not applicable |  | Not applicable |
|  |  |  |  |  |

For children, young- and old-old groups, estimates were obtained from a linear time trend model. For adults, estimates were obtained from a quadratic time trend model.

Each model was adjusted for categorical year, 5-year interval age, gender, marital status and 9-group prefecture. In analysis of children, marital status was not included. All analyses were adjusted for data clustering by household and weighted.

Back to the contents list (p.1)

## TABLE A9 Age-standardized prevalence rates and 95% confidence intervals for wellbeing and poor health by age-group and gender, 1986-2013

|  |  | **Wellbeing** | | | | |  | **Poor health** | |  |
| --- | --- | --- | --- | --- | --- | --- | --- | --- | --- | --- |
|  |  |  | | | | |  |  | |  |
| **Year** |  | **Men** | **Women** | | | |  | **Men** | **Women** |  |
|  |  |  |  | | | |  |  |  |  |
| **Children (6 to 19 years)** | | | | | | | | | | |
|  | | | |  |  | | | | | |
| 1989 |  | 67 (66, 68) | 65 (64, 66) | | | |  | 3 (2, 3) | 3 (3, 3) |  |
| 1992 |  | 71 (71, 72) | 70 (69, 71) | | | |  | 3 (2, 3) | 3 (2, 3) |  |
| 1995 |  | 71 (70, 72) | 69 (68, 70) | | | |  | 2 (2, 3) | 3 (2, 3) |  |
| 1998 |  | 64 (63, 66) | 63 (62, 64) | | | |  | 3 (3, 4) | 4 (3, 4) |  |
| 2001 |  | 63 (62, 65) | 62 (60, 63) | | | |  | 4 (3, 4) | 4 (3, 4) |  |
| 2004 |  | 67 (65, 68) | 64 (63, 66) | | | |  | 3 (2, 3) | 4 (3, 5) |  |
| 2007 |  | 64 (63, 66) | 62 (61, 64) | | | |  | 4 (3, 4) | 4 (3, 4) |  |
| 2010 |  | 64 (62, 66) | 62 (60, 64) | | | |  | 4 (3, 5) | 3 (2, 4) |  |
| 2013 |  | 67 (65, 68) | 66 (65, 68) | | | |  | 3 (2, 3) | 3 (2, 4) |  |
|  |  |  |  | | | |  |  |  |  |
| **Adults (20 to 59 years)** | | | | | | | | | | |
|  | | | |  |  | | | | | |
| 1986 |  | 43 (43, 44) | 37 (37, 38) | | | |  | 10 (9, 10) | 12 (11, 12) |  |
| 1989 |  | 49 (48, 49) | 43 (42, 43) | | | |  | 9 (9, 9) | 11 (11, 12) |  |
| 1992 |  | 53 (52, 54) | 48 (47, 49) | | | |  | 8 (8, 8) | 10 (9, 10) |  |
| 1995 |  | 54 (54, 55) | 50 (49, 50) | | | |  | 7 (7, 7) | 8 (8, 9) |  |
| 1998 |  | 48 (47, 49) | 44 (44, 45) | | | |  | 8 (8, 9) | 11 (10, 11) |  |
| 2001 |  | 46 (45, 46) | 41 (41, 42) | | | |  | 9 (9, 9) | 11 (11, 12) |  |
| 2004 |  | 46 (45, 47) | 43 (43, 44) | | | |  | 10 (9, 10) | 11 (11, 12) |  |
| 2007 |  | 40 (39, 41) | 38 (37, 39) | | | |  | 11 (10, 11) | 12 (12, 13) |  |
| 2010 |  | 39 (38, 40) | 37 (36, 38) | | | |  | 11 (10, 12) | 12 (12, 13) |  |
| 2013 |  | 40 (39, 41) | 39 (38, 39) | | | |  | 9 (9, 10) | 11 (11, 12) |  |
|  |  |  |  | | | |  |  |  |  |
| **Young-old (60 to 69 years)** | | | | | | | | | | |
|  |  |  | | | |  | | | |  |
| 1986 |  | 35 (33, 37) | 28 (26, 29) | | | |  | 17 (16, 18) | 21 (20, 22) |  |
| 1989 |  | 36 (35, 37) | 30 (29, 31) | | | |  | 17 (16, 18) | 20 (19, 21) |  |
| 1992 |  | 41 (39, 42) | 34 (33, 35) | | | |  | 16 (15, 17) | 17 (16, 18) |  |
| 1995 |  | 42 (41, 44) | 36 (35, 37) | | | |  | 14 (13, 15) | 16 (15, 17) |  |
| 1998 |  | 37 (36, 39) | 31 (30, 32) | | | |  | 15 (14, 16) | 17 (16, 18) |  |
| 2001 |  | 36 (35, 38) | 32 (30, 33) | | | |  | 16 (15, 17) | 18 (17, 19) |  |
| 2004 |  | 37 (36, 39) | 33 (31, 34) | | | |  | 17 (15, 18) | 18 (16, 19) |  |
| 2007 |  | 30 (29, 32) | 27 (26, 29) | | | |  | 18 (17, 19) | 19 (18, 20) |  |
| 2010 |  | 30 (29, 32) | 28 (27, 30) | | | |  | 16 (14, 17) | 17 (16, 18) |  |
| 2013 |  | 31 (29, 32) | 31 (30, 32) | | | |  | 14 (13, 15) | 14 (13, 15) |  |
|  |  |  |  | | | |  |  |  |  |
| **Old-old (70 to 79 years)** | | | | | | | | | | |
|  |  |  | | | |  | | | |  |
| 1986 |  | 30 (28, 32) | 22 (20, 23) | | | |  | 26 (24, 28) | 30 (28, 31) |  |
| 1989 |  | 31 (29, 33) | 24 (23, 26) | | | |  | 25 (23, 27) | 29 (27, 30) |  |
| 1992 |  | 35 (33, 36) | 29 (28, 31) | | | |  | 22 (20, 24) | 24 (23, 26) |  |
| 1995 |  | 38 (36, 40) | 31 (30, 33) | | | |  | 20 (18, 21) | 21 (20, 22) |  |
| 1998 |  | 34 (32, 36) | 28 (27, 30) | | | |  | 21 (20, 23) | 26 (24, 27) |  |
| 2001 |  | 31 (30, 33) | 27 (26, 29) | | | |  | 25 (23, 26) | 27 (25, 28) |  |
| 2004 |  | 29 (28, 31) | 26 (24, 27) | | | |  | 26 (24, 28) | 28 (26, 29) |  |
| 2007 |  | 26 (24, 28) | 19 (18, 21) | | | |  | 28 (26, 30) | 29 (27, 30) |  |
| 2010 |  | 28 (26, 30) | 22 (20, 24) | | | |  | 24 (22, 26) | 27 (25, 29) |  |
| 2013 |  | 28 (26, 30) | 25 (23, 26) | | | |  | 23 (22, 25) | 23 (22, 25) |  |
|  |  |  |  | | | |  |  |  |  |

Direct standardization method was used.

Back to the contents list (p.1)

## TABLE A10 The ratio of the prevalence proportions of wellbeing and poor health in 2013 compared with 1992, by age-group and gender

|  | **Model 1** | **Model 2** |
| --- | --- | --- |
|  |  |  |
| **Men** |  |  |
|  |  |  |
| **Wellbeing** | **Risk ratio (95% CI), p-value** | **Risk ratio (95% CI), p-value** |
|  |  |  |
| Children (6 to 18 years) | 0.943 (0.911, 0.975), 0.001 | 0.940 (0.908, 0.973), <0.001 |
| Adults (20 to 59 years) | 0.758 (0.736, 0.780), <0.001 | 0.761 (0.739, 0.785), <0.001 |
| Young-old (60 to 69 years) | 0.766 (0.722, 0.814), <0.001 | 0.777 (0.730, 0.826), <0.001 |
| Old-old (70 to 79 years) | 0.811 (0.746, 0.882), <0.001 | 0.815 (0.749, 0.887), <0.001 |
|  |  |  |
| **Poor health** |  |  |
|  |  |  |
| Children (6 to 18 years) | 0.982 (0.752, 1.282), 0.893 | 0.998 (0.766, 1.302), 0.989 |
| Adults (20 to 59 years) | 1.202 (1.113, 1.299), <0.001 | 1.149 (1.062, 1.244), 0.001 |
| Young-old (60 to 69 years) | 0.901 (0.811, 1.001), 0.052 | 0.831 (0.745, 0.927), 0.001 |
| Old-old (70 to 79 years) | 1.086 (0.979, 1.204), 0.118 | 1.064 (0.958, 1.181), 0.246 |
|  |  |  |
| **Women** |  |  |
|  |  |  |
| **Wellbeing** |  |  |
| Children (6 to 18 years) | 0.969 (0.937, 1.002), 0.064 | 0.965 (0.933, 0.998), 0.037 |
| Adults (20 to 59 years) | 0.809 (0.785, 0.833), <0.001 | 0.816 (0.791, 0.841), <0.001 |
| Young-old (60 to 69 years) | 0.909 (0.857, 0.965), 0.002 | 0.919 (0.865, 0.976), 0.006 |
| Old-old (70 to 79 years) | 0.848 (0.782, 0.919), <0.001 | 0.851 (0.783, 0.925), <0.001 |
|  |  |  |
| **Poor health** |  |  |
|  |  |  |
| Children (6 to 18 years) | 1.135 (0.869, 1.482), 0.353 | 1.169 (0.892, 1.532), 0.257 |
| Adults (20 to 59 years) | 1.172 (1.096, 1.254), <0.001 | 1.126 (1.052, 1.206), 0.001 |
| Young-old (60 to 69 years) | 0.835 (0.758, 0.921), <0.001 | 0.803 (0.729, 0.886), <0.001 |
| Old-old (70 to 79 years) | 0.967 (0.886, 1.055), 0.453 | 0.940 (0.860, 1.027), 0.170 |
|  |  |  |

Model 1: Adjusted for 5-year interval age

Model 2: Adjusted for 5-year interval age, 9-group prefecture and marital status. Marital status was not included in children

All analyses were adjusted for data clustering by household and weighted.

Back to the contents list (p.1)

## TABLE A11 Slope and Relative Indices of Inequality in wellbeing based on household income after tax by age-group and gender, 1986-2013

|  |  | **Slope Index of Inequality**  **(95% CI)** | | |  | **Relative Index of Inequality**  **(95% CI)** | |
| --- | --- | --- | --- | --- | --- | --- | --- |
|  |  |  | | |  |  | |
| **Year** |  | **Men** | **Women** | |  | **Men** | **Women** |
|  |  |  |  | |  |  |  |
| **Children (6 to 19 years)** | | | | | | | |
|  |  |  | |  | | | |
| 1989 |  | -0.07 (-0.11, -0.04) | -0.08 (-0.11, -0.04) | |  | 0.89 (0.84, 0.94) | 0.88 (0.83, 0.93) |
| 1992 |  | -0.11 (-0.14, -0.07) | -0.08 (-0.11, -0.04) | |  | 0.86 (0.81, 0.91) | 0.89 (0.84, 0.94) |
| 1995 |  | -0.06 (-0.10, -0.02) | -0.08 (-0.13, -0.04) | |  | 0.92 (0.87, 0.97) | 0.88 (0.83, 0.93) |
| 1998 |  | -0.01 (-0.06, 0.04) | -0.04 (-0.09, 0.00) | |  | 0.98 (0.91, 1.06) | 0.93 (0.86, 1.00) |
| 2001 |  | -0.05 (-0.11, 0.01) | 0.01 (-0.05, 0.06) | |  | 0.93 (0.85, 1.01) | 1.00 (0.91, 1.09) |
| 2004 |  | -0.01 (-0.08, 0.05) | -0.05 (-0.12, 0.02) | |  | 0.95 (0.86, 1.05) | 0.92 (0.83, 1.03) |
| 2007 |  | -0.06 (-0.13, 0.01) | -0.08 (-0.16, -0.01) | |  | 0.90 (0.81, 1.01) | 0.88 (0.78, 0.98) |
| 2010 |  | -0.13 (-0.21, -0.05) | -0.05 (-0.13, 0.03) | |  | 0.83 (0.73, 0.94) | 0.91 (0.81, 1.03) |
| 2013 |  | -0.11 (-0.18, -0.04) | -0.09 (-0.16, -0.02) | |  | 0.86 (0.78, 0.96) | 0.90 (0.81, 1.01) |
|  |  |  |  | |  |  |  |
| **Adults (20 to 59 years)** | | | | | | | |
|  |  |  | |  | | | |
| 1986 |  | -0.07 (-0.10, -0.05) | -0.05 (-0.07, -0.03) | |  | 0.85 (0.80, 0.89) | 0.88 (0.83, 0.93) |
| 1989 |  | -0.05 (-0.07, -0.03) | -0.06 (-0.08, -0.04) | |  | 0.91 (0.87, 0.95) | 0.88 (0.83, 0.92) |
| 1992 |  | -0.09 (-0.11, -0.07) | -0.10 (-0.13, -0.08) | |  | 0.85 (0.81, 0.89) | 0.81 (0.77, 0.84) |
| 1995 |  | -0.06 (-0.08, -0.04) | -0.08 (-0.10, -0.05) | |  | 0.90 (0.86, 0.94) | 0.86 (0.82, 0.90) |
| 1998 |  | -0.06 (-0.09, -0.03) | -0.07 (-0.10, -0.04) | |  | 0.89 (0.85, 0.94) | 0.86 (0.81, 0.91) |
| 2001 |  | -0.04 (-0.07, -0.01) | -0.05 (-0.08, -0.02) | |  | 0.93 (0.87, 0.99) | 0.88 (0.82, 0.94) |
| 2004 |  | -0.07 (-0.11, -0.03) | -0.05 (-0.08, -0.01) | |  | 0.86 (0.79, 0.93) | 0.90 (0.82, 0.98) |
| 2007 |  | -0.08 (-0.12, -0.04) | -0.07 (-0.11, -0.04) | |  | 0.82 (0.74, 0.90) | 0.83 (0.75, 0.92) |
| 2010 |  | -0.08 (-0.12, -0.04) | -0.09 (-0.13, -0.05) | |  | 0.80 (0.71, 0.89) | 0.78 (0.70, 0.87) |
| 2013 |  | -0.08 (-0.11, -0.04) | -0.10 (-0.14, -0.06) | |  | 0.82 (0.75, 0.90) | 0.77 (0.70, 0.84) |
|  |  |  |  | |  |  |  |
| **Young-old (60 to 69 years)** | | | | | | | |
|  |  |  | |  | | | |
| 1986 |  | -0.09 (-0.15, -0.04) | -0.04 (-0.09, 0.00) | |  | 0.77 (0.65, 0.90) | 0.86 (0.73, 1.02) |
| 1989 |  | -0.06 (-0.11, -0.01) | -0.06 (-0.10, -0.01) | |  | 0.84 (0.74, 0.96) | 0.83 (0.73, 0.96) |
| 1992 |  | -0.12 (-0.17, -0.07) | -0.06 (-0.10, -0.01) | |  | 0.74 (0.65, 0.83) | 0.85 (0.75, 0.96) |
| 1995 |  | -0.12 (-0.17, -0.07) | -0.05 (-0.09, 0.00) | |  | 0.76 (0.68, 0.86) | 0.88 (0.78, 1.00) |
| 1998 |  | -0.08 (-0.13, -0.03) | -0.07 (-0.12, -0.03) | |  | 0.80 (0.70, 0.91) | 0.79 (0.69, 0.92) |
| 2001 |  | -0.12 (-0.17, -0.06) | -0.04 (-0.09, 0.02) | |  | 0.72 (0.62, 0.84) | 0.88 (0.75, 1.04) |
| 2004 |  | -0.08 (-0.15, -0.02) | 0.00 (-0.07, 0.06) | |  | 0.80 (0.66, 0.96) | 0.98 (0.81, 1.19) |
| 2007 |  | -0.15 (-0.21, -0.09) | -0.07 (-0.14, -0.01) | |  | 0.60 (0.48, 0.75) | 0.76 (0.61, 0.96) |
| 2010 |  | -0.06 (-0.12, 0.00) | -0.05 (-0.12, 0.01) | |  | 0.83 (0.68, 1.02) | 0.83 (0.67, 1.02) |
| 2013 |  | -0.10 (-0.15, -0.04) | -0.05 (-0.10, 0.01) | |  | 0.73 (0.60, 0.89) | 0.86 (0.72, 1.02) |
|  |  |  |  | |  |  |  |
| **Old-old (70 to 79 years)** | | | | | | | |
|  |  |  | |  | | | |
| 1986 |  | 0.00 (-0.08, 0.07) | 0.05 (-0.01, 0.10) | |  | 0.99 (0.78, 1.26) | 1.22 (0.96, 1.54) |
| 1989 |  | -0.13 (-0.20, -0.06) | 0.00 (-0.05, 0.05) | |  | 0.66 (0.53, 0.82) | 1.01 (0.83, 1.25) |
| 1992 |  | -0.06 (-0.13, 0.01) | -0.03 (-0.08, 0.03) | |  | 0.84 (0.69, 1.03) | 0.91 (0.76, 1.09) |
| 1995 |  | 0.03 (-0.05, 0.10) | 0.04 (-0.01, 0.10) | |  | 1.06 (0.88, 1.28) | 1.16 (0.97, 1.38) |
| 1998 |  | -0.06 (-0.13, 0.01) | 0.01 (-0.04, 0.06) | |  | 0.85 (0.69, 1.04) | 1.04 (0.87, 1.24) |
| 2001 |  | -0.06 (-0.13, 0.01) | -0.04 (-0.09, 0.02) | |  | 0.81 (0.65, 1.01) | 0.87 (0.72, 1.06) |
| 2004 |  | -0.09 (-0.16, -0.02) | 0.00 (-0.06, 0.06) | |  | 0.74 (0.57, 0.95) | 0.98 (0.78, 1.23) |
| 2007 |  | -0.11 (-0.18, -0.04) | -0.02 (-0.08, 0.03) | |  | 0.65 (0.49, 0.85) | 0.88 (0.66, 1.17) |
| 2010 |  | -0.11 (-0.18, -0.03) | 0.00 (-0.06, 0.06) | |  | 0.67 (0.51, 0.88) | 0.98 (0.75, 1.29) |
| 2013 |  | -0.09 (-0.16, -0.02) | -0.06 (-0.11, 0.00) | |  | 0.73 (0.58, 0.94) | 0.79 (0.63, 0.99) |
|  |  |  |  | |  |  |  |

Estimates were calculated by each survey year separately and adjusted for 5-year interval age, marital status, 9-group prefecture. All analyses were adjusted for data clustering by household and weighted. In analysis of children, marital status was not included.

Back to the contents list (p.1)

## TABLE A12 Time trends for the Slope and Relative Indices of Inequality in wellbeing based on household income after tax by age-group and gender, 1992-2013

|  |  | **Male** | **Female** |
| --- | --- | --- | --- |
|  |  |  |  |
|  |  | **Coefficient (95% CI), p-value** | **Coefficient (95% CI), p-value** |
|  |  |  |  |
|  | **Slope Index of Inequality** | | |
|  |  | | |
| **Children (6 to 19 years)** |  |  |  |
|  |  |  |  |
| Main effect (in 1992) |  | -0.101 (-0.133, -0.069), <0.001 | -0.089 (-0.122, -0.057), <0.001 |
| Linear trend term |  | 0.016 (0.0067, 0.025), 0.001 | 0.011 (0.002, 0.020), 0.021 |
| Quadratic trend term |  | -0.00084 (-0.0013, -0.00037), <0.001 | -0.00052 (-0.00099, -0.000062), 0.026 |
|  |  |  |  |
| **Adults (20 to 59 years)** |  |  |  |
|  |  |  |  |
| Main effect (in 1992) |  | -0.086 (-0.105, -0.066), <0.001 | -0.106 (-0.125, -0.087), <0.001 |
| Linear trend term |  | 0.0062 (0.0010, 0.011), 0.019 | 0.0095 (0.0045, 0.014), <0.001 |
| Quadratic trend term |  | -0.00032 (-0.00057, -0.000063), 0.015 | -0.00045 (-0.00069, -0.00021), <0.001 |
|  |  |  |  |
| **Young-old** **(60 to 69 years)** |  |  |  |
|  |  |  |  |
| Main effect (in 1992) |  | -0.108 (-0.140, -0.077), <0.001 | -0.051 (-0.080, -0.022), 0.001 |
| Linear trend term |  | 0.00043 (-0.0023, 0.0031), 0.752 | 0.00023 (-0.0023, 0.0028), 0.860 |
| Quadratic trend term |  | Not applicable | Not applicable |
|  |  |  |  |
| **Old-old (70 to 79 years)** |  |  |  |
|  |  |  |  |
| Main effect (in 1992) |  | -0.028 (-0.073, 0.016), 0.212 | 0.017 (-0.017, 0.051), 0.327 |
| Linear trend term |  | -0.0037 (-0.0072, -0.00014), 0.042 | -0.0029 (-0.0056, -0.00012), 0.042 |
| Quadratic trend term |  | Not applicable | Not applicable |
|  |  |  |  |
|  | **Relative Index of Inequality** | | |
|  |  | | |
| **Children (6 to 19 years)** |  |  |  |
|  |  |  |  |
| Main effect (in 1992) |  | 0.864 (0.825, 0.905), <0.001 | 0.895 (0.859, 0.931), <0.001 |
| Linear trend term |  | 1.021 (1.008, 1.035), 0.002 | 1.002 (0.998, 1.006), 0.407 |
| Quadratic trend term |  | 0.999 (0.998, 1.000), 0.002 | Not applicable |
|  |  |  |  |
| **Adults (20 to 59 years)** |  |  |  |
|  |  |  |  |
| Main effect (in 1992) |  | 0.845 (0.813, 0.879), <0.001 | 0.802 (0.770, 0.834), <0.001 |
| Linear trend term |  | 1.014 (1.003, 1.026), 0.011 | 1.020 (1.009, 1.032), 0.001 |
| Quadratic trend term |  | 0.999 (0.999, 1.000), 0.003 | 0.999 (0.998, 1.000), <0.001 |
|  |  |  |  |
| **Young-old** **(60 to 69 years)** |  |  |  |
|  |  |  |  |
| Main effect (in 1992) |  | 0.772 (0.711, 0.838), <0.001 | 0.863 (0.792, 0.940), 0.001 |
| Linear trend term |  | 0.996 (0.988, 1.004), 0.370 | 0.999 (0.992, 1.007), 0.883 |
| Quadratic trend term |  | Not applicable | Not applicable |
|  |  |  |  |
| **Old-old (70 to 79 years)** |  |  |  |
|  |  |  |  |
| Main effect (in 1992) |  | 0.933 (0.819, 1.063), 0.300 | 1.062 (0.946, 1.192), 0.306 |
| Linear trend term |  | 0.985 (0.974, 0.996), 0.010 | 0.989 (0.978, 0.999), 0.031 |
| Quadratic trend term |  | Not applicable | Not applicable |
|  |  |  |  |

Estimates were obtained from a quadratic time trend model. For the young- and old-old groups, as well as relative index of inequality for female children, estimates were obtained from a linear time trend model.

Each model was adjusted for categorical year, 5-year interval age, gender, marital status and 9-group prefecture. In analysis of children, marital status was not included. All analyses were adjusted for data clustering by household and weighted.

Back to the contents list (p.1)

## TABLE A13 The comparison of Slope and Relative Indices of Inequality in wellbeing based on household income after tax in 1992 and 2013, by age-group and gender

|  | **Male** | **Female** |
| --- | --- | --- |
|  |  |  |
|  | **Coefficient (95% CI), p-value** | **Coefficient (95% CI), p-value** |
|  |  |  |
| **Slope Index of Inequality** |  | |
|  |  | |
| Children (6 to 19 years) | 0.0027 (-0.077, 0.082), 0.948 | -0.0012 (-0.079, 0.077), 0.977 |
| Adults (20 to 59 years) | 0.00057 (-0.042, 0.044), 0.979 | 0.0025 (-0.038, 0.043), 0.905 |
| Young-old (60 to 69 years) | 0.010 (-0.062, 0.083), 0.782 | 0.019 (-0.049, 0.087), 0.581 |
| Old-old (70 to 79 years) | -0.043 (-0.138, 0.052), 0.373 | -0.053 (-0.127, 0.022), 0.164 |
|  |  |  |
| **Relative Index of Inequality** |  | |
|  |  | |
| Children (6 to 19 years) | 1.008 (0.897, 1.133), 0.892 | 1.013 (0.902, 1.138), 0.825 |
| Adults (20 to 59 years) | 0.963 (0.869, 1.067), 0.471 | 0.962 (0.869, 1.065), 0.456 |
| Young-old (60 to 69 years) | 0.943 (0.758, 1.173), 0.597 | 1.046 (0.851, 1.287), 0.667 |
| Old-old (70 to 79 years) | 0.840 (0.617, 1.142), 0.265 | 0.808 (0.614, 1.063), 0.128 |
|  |  |  |

Each model included an interaction between the rank variable for SII (or RII) and year (categorical, included only 1992 and 2013) and adjusted for 9-group prefecture, 5-year interval age and marital status. All analyses were adjusted for data clustering by household and weighted. Marital status was not included in children. 1992 is the reference, and the coefficients of an interaction term between the rank variable and year are reported in the table. The coefficients indicate the magnitude of change in SII in 2013 compared with 1992.

Back to the contents list (p.1)

## TABLE A14 Slope and Relative Indices of Inequality in poor health based on household income after tax by age-group and gender, 1986-2013

|  |  | **Slope Index of Inequality**  **(95% CI)** | | | |  | **Relative Index of Inequality**  **(95% CI)** | |
| --- | --- | --- | --- | --- | --- | --- | --- | --- |
|  |  |  | | | |  |  | |
| **Year** |  | **Men** | **Women** | | |  | **Men** | **Women** |
|  |  |  |  | | |  |  |  |
| **Children (6 to 19 years)** | | | | | | | | |
|  | | | |  |  | | | |
| 1989 |  | 0.00 (-0.01, 0.01) | 0.01 (0.00, 0.02) | | |  | 1.15 (0.78, 1.70) | 1.29 (0.88, 1.89) |
| 1992 |  | 0.01 (0.00, 0.02) | 0.00 (-0.01, 0.01) | | |  | 1.54 (0.96, 2.47) | 1.03 (0.66, 1.61) |
| 1995 |  | 0.00 (-0.01, 0.01) | 0.00 (-0.02, 0.01) | | |  | 0.96 (0.61, 1.51) | 0.94 (0.59, 1.52) |
| 1998 |  | 0.00 (-0.02, 0.01) | 0.01 (-0.01, 0.03) | | |  | 0.91 (0.54, 1.53) | 1.34 (0.84, 2.14) |
| 2001 |  | 0.00 (-0.02, 0.02) | 0.00 (-0.03, 0.02) | | |  | 0.92 (0.52, 1.61) | 0.90 (0.51, 1.58) |
| 2004 |  | 0.00 (-0.02, 0.03) | 0.03 (0.01, 0.06) | | |  | 1.12 (0.48, 2.61) | 2.21 (1.17, 4.17) |
| 2007 |  | -0.02 (-0.04, 0.01) | 0.01 (-0.02, 0.04) | | |  | 0.58 (0.29, 1.16) | 1.26 (0.64, 2.45) |
| 2010 |  | 0.03 (0.00, 0.06) | 0.00 (-0.02, 0.03) | | |  | 2.08 (1.03, 4.21) | 1.17 (0.51, 2.67) |
| 2013 |  | 0.00 (-0.02, 0.03) | 0.00 (-0.02, 0.03) | | |  | 1.14 (0.50, 2.61) | 1.06 (0.48, 2.34) |
|  |  |  |  | | |  |  |  |
| **Adults (20 to 59 years)** | | | | | | | | |
|  | | | |  |  | | | |
| 1986 |  | 0.05 (0.04, 0.06) | 0.04 (0.02, 0.05) | | |  | 1.68 (1.46, 1.93) | 1.38 (1.22, 1.55) |
| 1989 |  | 0.04 (0.03, 0.06) | 0.04 (0.03, 0.05) | | |  | 1.65 (1.44, 1.88) | 1.46 (1.30, 1.63) |
| 1992 |  | 0.06 (0.05, 0.07) | 0.05 (0.04, 0.06) | | |  | 2.22 (1.90, 2.59) | 1.71 (1.49, 1.95) |
| 1995 |  | 0.03 (0.02, 0.05) | 0.02 (0.01, 0.04) | | |  | 1.63 (1.37, 1.94) | 1.35 (1.15, 1.57) |
| 1998 |  | 0.05 (0.03, 0.06) | 0.04 (0.02, 0.05) | | |  | 1.76 (1.48, 2.09) | 1.40 (1.21, 1.63) |
| 2001 |  | 0.03 (0.02, 0.05) | 0.03 (0.01, 0.04) | | |  | 1.43 (1.18, 1.73) | 1.26 (1.07, 1.49) |
| 2004 |  | 0.06 (0.04, 0.09) | 0.05 (0.02, 0.07) | | |  | 1.88 (1.51, 2.33) | 1.51 (1.23, 1.84) |
| 2007 |  | 0.04 (0.02, 0.06) | 0.04 (0.02, 0.06) | | |  | 1.40 (1.14, 1.73) | 1.38 (1.14, 1.66) |
| 2010 |  | 0.07 (0.04, 0.09) | 0.06 (0.03, 0.09) | | |  | 1.77 (1.40, 2.23) | 1.60 (1.30, 1.98) |
| 2013 |  | 0.05 (0.03, 0.08) | 0.07 (0.05, 0.09) | | |  | 1.69 (1.33, 2.13) | 1.88 (1.52, 2.33) |
|  |  |  |  | | |  |  |  |
| **Young-old (60 to 69 years)** | | | | | | | | |
|  | | | |  |  | | | |
| 1986 |  | 0.10 (0.06, 0.15) | 0.06 (0.02, 0.11) | | |  | 1.83 (1.41, 2.38) | 1.37 (1.11, 1.69) |
| 1989 |  | 0.08 (0.04, 0.11) | 0.09 (0.06, 0.13) | | |  | 1.58 (1.26, 1.98) | 1.62 (1.34, 1.96) |
| 1992 |  | 0.12 (0.08, 0.15) | 0.10 (0.06, 0.13) | | |  | 2.12 (1.70, 2.65) | 1.81 (1.47, 2.23) |
| 1995 |  | 0.08 (0.05, 0.12) | 0.06 (0.03, 0.10) | | |  | 1.83 (1.41, 2.37) | 1.50 (1.19, 1.89) |
| 1998 |  | 0.10 (0.06, 0.14) | 0.08 (0.04, 0.12) | | |  | 1.91 (1.47, 2.47) | 1.60 (1.28, 2.01) |
| 2001 |  | 0.11 (0.07, 0.15) | 0.08 (0.03, 0.12) | | |  | 1.99 (1.53, 2.59) | 1.51 (1.19, 1.92) |
| 2004 |  | 0.12 (0.06, 0.17) | 0.04 (0.00, 0.09) | | |  | 2.02 (1.48, 2.75) | 1.29 (0.97, 1.71) |
| 2007 |  | 0.16 (0.11, 0.22) | 0.05 (0.00, 0.10) | | |  | 2.49 (1.83, 3.40) | 1.30 (0.98, 1.73) |
| 2010 |  | 0.12 (0.07, 0.17) | 0.13 (0.08, 0.17) | | |  | 2.28 (1.64, 3.15) | 2.12 (1.58, 2.84) |
| 2013 |  | 0.11 (0.07, 0.15) | 0.07 (0.02, 0.11) | | |  | 2.21 (1.62, 3.01) | 1.62 (1.20, 2.18) |
|  |  |  |  | | |  |  |  |
| **Old-old (70 to 79 years)** | | | | | | | | |
|  | | | |  |  | | | |
| 1986 |  | 0.11 (0.04, 0.18) | 0.01 (-0.05, 0.07) | | |  | 1.51 (1.14, 2.00) | 1.05 (0.85, 1.29) |
| 1989 |  | 0.14 (0.08, 0.20) | 0.07 (0.02, 0.12) | | |  | 1.74 (1.35, 2.25) | 1.29 (1.07, 1.55) |
| 1992 |  | 0.11 (0.05, 0.17) | 0.07 (0.02, 0.12) | | |  | 1.67 (1.27, 2.20) | 1.32 (1.07, 1.62) |
| 1995 |  | 0.04 (-0.02, 0.10) | 0.00 (-0.04, 0.05) | | |  | 1.21 (0.90, 1.64) | 1.02 (0.81, 1.29) |
| 1998 |  | 0.06 (0.00, 0.12) | 0.05 (0.00, 0.10) | | |  | 1.28 (0.96, 1.72) | 1.22 (1.00, 1.48) |
| 2001 |  | 0.16 (0.10, 0.22) | 0.05 (0.00, 0.11) | | |  | 1.83 (1.45, 2.32) | 1.22 (0.99, 1.49) |
| 2004 |  | 0.09 (0.02, 0.16) | -0.03 (-0.09, 0.03) | | |  | 1.41 (1.08, 1.83) | 0.88 (0.72, 1.09) |
| 2007 |  | 0.12 (0.05, 0.20) | 0.03 (-0.03, 0.09) | | |  | 1.53 (1.18, 1.98) | 1.10 (0.89, 1.37) |
| 2010 |  | 0.13 (0.06, 0.20) | 0.05 (-0.02, 0.11) | | |  | 1.68 (1.25, 2.26) | 1.19 (0.94, 1.52) |
| 2013 |  | 0.11 (0.05, 0.18) | 0.07 (0.01, 0.12) | | |  | 1.62 (1.23, 2.13) | 1.33 (1.06, 1.68) |
|  |  |  |  | | |  |  |  |

Estimates were calculated by each survey year separately and adjusted for 5-year interval age, marital status, 9-group prefecture. All analyses were adjusted for data clustering by household and weighted. In analysis of children, marital status was not included.

Back to the contents list (p.1)

## TABLE A15 Time trends for the Slope and Relative Indices of Inequality in poor health based on household income after tax by age-group and gender, 1992-2013

|  |  | **Male** | **Female** |
| --- | --- | --- | --- |
|  |  |  |  |
|  |  | **Coefficient (95% CI), p-value** | **Coefficient (95% CI), p-value** |
|  |  |  |  |
|  | | **Slope Index of Inequality** | |
|  | |  | |
| **Children (6 to 19 years)** |  |  |  |
|  |  |  |  |
| Main effect (in 1992) |  | 0.0040 (-0.0049, 0.013), 0.382 | 0.0026 (-0.0067, 0.012), 0.581 |
| Linear trend term |  | -0.00017 (-0.0011, 0.00077), 0.721 | 0.00041 (-0.00054, 0.0014), 0.400 |
| Quadratic trend term |  | Not applicable | Not applicable |
|  |  |  |  |
| **Adults (20 to 59 years)** |  |  |  |
|  |  |  |  |
| Main effect (in 1992) |  | 0.056 (0.045, 0.066), <0.001 | 0.044 (0.033, 0.055), <0.001 |
| Linear trend term |  | -0.0031 (-0.0060, -0.00016), 0.039 | -0.0038 (-0.0068, -0.00073), 0.015 |
| Quadratic trend term |  | 0.00017 (0.000019, 0.00031), 0.027 | 0.00025 (0.00010, 0.00041), 0.001 |
|  |  |  |  |
| **Young-old (60 to 69 years)** |  |  |  |
|  |  |  |  |
| Main effect (in 1992) |  | 0.095 (0.071, 0.118), <0.001 | 0.077 (0.054, 0.100), <0.001 |
| Linear trend term |  | 0.0017 (-0.00038, 0.0037), 0.109 | -0.00011 (-0.0021, 0.0019), 0.912 |
| Quadratic trend term |  | Not applicable | Not applicable |
|  |  |  |  |
| **Old-old (70 to 79 years)** |  |  |  |
|  |  |  |  |
| Main effect (in 1992) |  | 0.084 (0.046, 0.123), <0.001 | 0.033 (0.001, 0.065), 0.041 |
| Linear trend term |  | 0.0020 (-0.0012, 0.0052), 0.217 | 0.00070 (-0.0020, 0.0034), 0.610 |
| Quadratic trend term |  | Not applicable | Not applicable |
|  |  |  |  |
|  | | **Relative Index of Inequality** | |
|  | |  | |
| **Children (6 to 19 years)** |  |  |  |
|  |  |  |  |
| Main effect (in 1992) |  | 1.140 (0.830, 1.566), 0.419 | 1.060 (0.776, 1.450), 0.713 |
| Linear trend term |  | 0.995 (0.964, 1.026), 0.735 | 1.014 (0.983, 1.046), 0.387 |
| Quadratic trend term |  | Not applicable | Not applicable |
|  |  |  |  |
| **Adults (20 to 59 years)** |  |  |  |
|  |  |  |  |
| Main effect (in 1992) |  | 2.083 (1.825, 2.379), <0.001 | 1.642 (1.464, 1.842), <0.001 |
| Linear trend term |  | 0.950 (0.919, 0.982), 0.003 | 0.953 (0.925, 0.980), 0.001 |
| Quadratic trend term |  | 1.002 (1.001, 1.004), 0.010 | 1.003 (1.001, 1.004), <0.001 |
|  |  |  |  |
| **Young-old (60 to 69 years)** |  |  |  |
|  |  |  |  |
| Main effect (in 1992) |  | 1.856 (1.587, 2.170), <0.001 | 1.778 (1.487, 2.125), <0.001 |
| Linear trend term |  | 1.011 (0.997, 1.025), 0.110 | 0.958 (0.917, 1.000), 0.052 |
| Quadratic trend term |  | Not applicable | 1.002 (1.000, 1.004), 0.044 |
|  |  |  |  |
| **Old-old (70 to 79 years)** |  |  |  |
|  |  |  |  |
| Main effect (in 1992) |  | 1.470 (1.233, 1.754), <0.001 | 1.289 (1.085, 1.532), 0.004 |
| Linear trend term |  | 1.005 (0.991, 1.019), 0.508 | 0.964 (0.928, 1.002), 0.063 |
| Quadratic trend term |  | Not applicable | 1.002 (1.000, 1.004), 0.046 |
|  |  |  |  |

Estimates were obtained from a quadratic time trend model for adults and female young- and old-old in Relative Index of Inequality. For other age groups, estimates were obtained from a linear time trend model.

Each model was adjusted for categorical year, 5-year interval age, gender, marital status and 9-group prefecture. The last four variables were included with their interactions with year. In analysis of children, marital status was not included. All analyses were adjusted for data clustering by household and weighted.

Back to the contents list (p.1)

## TABLE A16 The comparison of Slope and Relative Indices of Inequality in poor health based on household income after tax in 1992 and 2013, by age-group and gender

|  | **Male** | **Female** |  |
| --- | --- | --- | --- |
|  |  |  |  |
|  | **Coefficient (95% CI), p-value** | **Coefficient (95% CI), p-value** |  |
|  |  |  |  |
| **Slope Index of Inequality** |  | | |
|  |  | | |
| Children (6 to 19 years) | -0.010 (-0.036, 0.016), 0.455 | 0.00011 (-0.027, 0.027), 0.993 |  |
| Adults (20 to 59 years) | -0.0072 (-0.033, 0.018), 0.582 | 0.024 (-0.003, 0.050), 0.079 |  |
| Young-old (60 to 69 years) | 0.001 (-0.054, 0.056), 0.973 | -0.031 (-0.082, 0.020), 0.238 |  |
| Old-old (70 to 79 years) | 0.0022 (-0.083, 0.088), 0.959 | 0.0040 (-0.067, 0.075), 0.913 |  |
|  |  |  |  |
| **Relative Index of Inequality** |  | | |
|  |  | | |
| Children (6 to 19 years) | 0.696 (0.264, 1.834), 0.464 | 1.008 (0.403, 2.519), 0.986 |  |
| Adults (20 to 59 years) | 0.799 (0.607, 1.052), 0.110 | 1.131 (0.888, 1.441), 0.319 |  |
| Young-old (60 to 69 years) | 1.113 (0.765, 1.620), 0.575 | 0.913 (0.644, 1.293), 0.607 |  |
| Old-old (70 to 79 years) | 0.973 (0.668, 1.418), 0.889 | 1.036 (0.764, 1.404), 0.821 |  |
|  |  |  |  |

Each model included an interaction between the rank variable for SII (or RII) and year (categorical, included only 1992 and 2013) and adjusted for 9-group prefecture, 5-year interval age and marital status. All analyses were adjusted for data clustering by household and weighted. Marital status was not included in children. 1992 is the reference, and the coefficients of an interaction term between the rank variable and year are reported in the table. The coefficients indicate the magnitude of change in SII in 2013 compared with 1992.

Back to the contents list (p.1)

## TABLE A17 Interaction of the Slope Index of Inequality in wellbeing and poor health based on income after tax with employment status, 1992-2013

|  | **Wellbeing** | **Poor health** |
| --- | --- | --- |
|  |  |  |
|  | **Coefficient (95% CI), p-value** | **Coefficient (95% CI), p-value** |
|  |  |  |
|  | **Adults (20 to 59 years)** | |
|  |  |  |
| SII (main effect) | -0.085 (-0.102, -0.068), <0.001 | 0.038 (0.030, 0.047), <0.001 |
| Linear trend | 0.0082 (0.0040, 0.012), <0.001 | -0.0045 (-0.0068, -0.0022), <0.001 |
| Quadratic trend | -0.00038 (-0.00059, -0.00017), <0.001 | 0.00021 (0.000094, 0.00033), <0.001 |
| Employment | -0.0029 (-0.014, 0.0077), 0.587 | 0.0088 (0.0013, 0.014), 0.017 |
| SII*employment | -0.034 (-0.054, -0.014), 0.001 | 0.028 (0.015, 0.041), <0.001 |
| Linear trend*employment | -0.0010 (-0.0048, 0.0027), 0.583 | 0.0041 (0.0014, 0.0067), 0.003 |
| Quadratic trend*employment | 0.000043 (-0.00014, 0.00023), 0.652 | -0.000062 (-0.00020, 0.000075), 0.376 |
|  |  |  |
|  | **Young-old (60 to 69 years)** | |
|  |  |  |
| SII (main effect) | -0.045 (-0.082, -0.0080), 0.017 | 0.055 (0.030, 0.080), <0.001 |
| Linear trend | -0.0013 (-0.010, 0.0072), 0.763 | -0.0031 (-0.0091, 0.0030), 0.318 |
| Quadratic trend | -0.000024 (-0.00042, 0.00038), 0.908 | 0.00016 (-0.00012, 0.00044), 0.272 |
| Employment | -0.052 (-0.069, -0.036), <0.001 | 0.043 (0.031, 0.054), <0.001 |
| SII*employment | -0.036 (-0.074, 0.00062), 0.054 | 0.048 (0.020, 0.076), 0.001 |
| Linear trend*employment | 0.00092 (-0.0060, 0.0078), 0.795 | 0.0022 (-0.0033, 0.0076), 0.436 |
| Quadratic trend*employment | 0.00015 (-0.00018, 0.00047), 0.368 | -0.00014 (-0.00039, 0.00012), 0.290 |
|  |  |  |
|  | **Old-old (70 to 79 years)** | |
|  |  |  |
| SII (main effect) | 0.010 (-0.049, 0.069), 0.747 | 0.067 (0.019, 0.114), 0.006 |
| Linear trend | -0.0054 (-0.017, 0.0066), 0.382 | -0.0020 (-0.012, 0.0083), 0.698 |
| Quadratic trend | -0.000015 (-0.00057, 0.00054), 0.959 | 0.000083 (-0.00040, 0.00056), 0.733 |
| Employment | -0.074 (-0.096, -0.052), <0.001 | 0.077 (0.059, 0.095), <0.001 |
| SII*employment | -0.012 (-0.069, 0.045), 0.681 | -0.010 (-0.057, 0.038), 0.692 |
| Linear trend*employment | 0.0054 (-0.0051, 0.016), 0.314 | -0.0020 (-0.011, 0.0073), 0.711 |
| Quadratic trend*employment | -0.000097 (-0.00581, 0.00039), 0.695 | 0.00013 (-0.00028, 0.00055), 0.527 |
|  |  |  |

Employment is coded as 0=employed, 1=not employed.

Each model was adjusted for categorical year, 5-year interval age, gender, marital status and 9-group prefecture. All analyses were adjusted for data clustering by household and weighted.

Back to the contents list (p.1)

## TABLE A18 The ratio of the prevalence proportions of wellbeing and poor health in 2013 compared with 1992 for children excluding aged 6-11 years

|  | **Model 1** | **Model 2** |
| --- | --- | --- |
|  |  |  |
| **Wellbeing** | **Risk ratio (95% CI), p-value** | **Risk ratio (95% CI), p-value** |
|  |  |  |
| Children (12 to 19 years) | 0.873 (0.842, 0.905), <0.001 | 0.870 (0.840, 0.902), <0.001 |
|  |  |  |
| **Poor health** |  |  |
|  |  |  |
| Children (12 to 19 years) | 1.377 (1.118, 1.697), 0.003 | 1.395 (1.131, 1.722), 0.002 |
|  |  |  |

Model 1: Adjusted for 5-year interval age and gender

Model 2: Adjusted for 5-year interval age, gender and 9-group prefecture.

All analyses were adjusted for data clustering by household and weighted.

Back to the contents list (p.1)

## TABLE A19 Slope Index of Inequality for wellbeing and poor health in 2013 compared with 1992 for children excluding aged 6-11 years

|  | **Coefficient (95% CI), p-value** |
| --- | --- |
|  |  |
| **Slope Index of Inequality** |  |
| **Wellbeing** |  |
|  |  |
| Children (12 to 19 years) | -0.048 (-0.121, 0.026), 0.202 |
|  |  |
| **Poor health** |  |
|  |  |
| Children (12 to 19 years) | -0.012 (-0.039, 0.015), 0.383 |
|  |  |

Each model included an interaction between the rank variable for SII (or RII) and year (categorical, included only 1992 and 2013) and adjusted for 9-group prefecture and 5-year interval age. All analyses were adjusted for data clustering by household and weighted. 1992 is the reference, and the coefficients of an interaction term between the rank variable and year are reported in the table. The coefficients indicate the magnitude of change in SII (or RII) in 2013 compared with 1992.

Back to the contents list (p.1)

## TABLE A20 The ratio of the prevalence proportion of wellbeing and poor health in 2013 compared with 1986, and the Slope and Relative Indices of Inequality in wellbeing and poor health in 2013 compared with 1986, by age-group

|  | **Wellbeing** | **Poor health** |
| --- | --- | --- |
|  |  |  |
|  | **Coefficient (95% CI), p-value** | **Coefficient (95% CI), p-value** |
|  |  |  |
| **Ratio of the prevalence proportion** |  |  |
|  |  |  |
| Children (6 to 18 years) | 1.019 (0.992, 1.046), 0.173 | 0.987 (0.817, 1.191), 0.891 |
| Adults (20 to 59 years) | 0.992 (0.967, 1.017), 0.528 | 0.921 (0.873, 0.971), 0.002 |
| Young-old (60 to 69 years) | 1.003 (0.952, 1.057), 0.913 | 0.704 (0.653, 0.760), <0.001 |
| Old-old (70 to 79 years) | 1.022 (0.950, 1.100), 0.561 | 0.828 (0.771, 0.889), <0.001 |
|  |  |  |
| **Slope Index of Inequality** |  | |
|  |  | |
| Children (6 to 19 years) | -0.010 (-0.071, 0.051), 0.744 | -0.004 (-0.023, 0.014), 0.652 |
| Adults (20 to 59 years) | -0.031 (-0.064, 0.002), 0.065 | 0.020 (0.000, 0.039), 0.052 |
| Young-old (60 to 69 years) | 0.013 (-0.042, 0.067), 0.654 | -0.00023 (-0.043, 0.043), 0.992 |
| Old-old (70 to 79 years) | -0.092 (-0.156, -0.029), 0.004 | 0.015 (-0.047, 0.077), 0.632 |
|  |  |  |

Children was compared with 1989.

Ratio of the prevalence proportions was adjusted for 5-year interval age, gender, 9-group prefecture and marital status. Marital status was not included in children (equivalent to Model 2 in Table 2).

For the analysis of SII and RII, each model included an interaction between the rank variable for SII and year (categorical, included only 1986 (or 1989) and 2013) and adjusted for 5-year interval age, gender, marital status and 9-group prefecture. Marital status was not included in children. 1986 (or 1989) is the reference, and the coefficients of an interaction term between the rank variable and year are reported in the table. The coefficients indicate the magnitude of change in SII in 2013 compared with 1986 (or 1989).

Back to the contents list (p.1)

## TABLE A21 The ratio of the prevalence proportions of wellbeing and poor health in 2013 compared with 1992, by age-group, using sample after multilevel multiple imputation

|  | **Model 1** | **Model 2** |
| --- | --- | --- |
|  |  |  |
| **Wellbeing** | **Risk ratio (95% CI), p-value** | **Risk ratio (95% CI), p-value** |
|  |  |  |
| Children (6 to 19 years) | 0.96 (0.94, 0.98), <0.001 | 0.96 (0.94, 0.98), <0.001 |
| Adults (20 to 59 years) | 0.79 (0.77, 0.80), <0.001 | 0.79 (0.78, 0.80), <0.001 |
| Young-old (60 to 69 years) | 0.80 (0.77, 0.83), <0.001 | 0.81 (0.78, 0.84), <0.001 |
| Old-old (70 to 79 years) | 0.80 (0.77, 0.85), <0.001 | 0.81 (0.77, 0.85), <0.001 |
|  |  |  |
| **Poor health** |  |  |
|  |  |  |
| Children (6 to 19 years) | 0.99 (0.86, 1.14), 0.887 | 1.02 (0.88, 1.18), 0.773 |
| Adults (20 to 59 years) | 1.15 (1.11, 1.20), <0.001 | 1.12 (1.08, 1.17), <0.001 |
| Young-old (60 to 69 years) | 0.92 (0.87, 0.97), 0.004 | 0.88 (0.83, 0.93), <0.001 |
| Old-old (70 to 79 years) | 1.06 (1.00, 1.12), 0.039 | 1.04 (0.98, 1.11), 0.152 |
|  |  |  |

Analyses in this table did not use imputed data because all covariates did not have missing data and observations with imputed outcome were not analysed. Increased sample sizes reflect increased complete cases due to multiple imputation to missing data in household income.

Sample size was 29,550 for children, 89,310 for adults, 22,381 for Young-old, and 14,161 for old-old.

Model 1: Adjusted for 5-year interval age and gender

Model 2: Adjusted for 5-year interval age, gender, 9-group prefecture and marital status.

Marital status was not included in children

All analyses were adjusted for data clustering by household.

Back to the contents list (p.1)

## TABLE A22 Slope Index of Inequality for wellbeing and poor health in 2013 compared with 1992, by age-group, using sample after multilevel multiple imputation

|  | **Slope Index of Inequality (95% CI), p-value** |
| --- | --- |
|  |  |
| **Wellbeing** |  |
|  |  |
| Children (6 to 19 years) | 0.015 (-0.036, 0.065), 0.569 |
| Adults (20 to 59 years) | 0.017 (-0.009, 0.043), 0.209 |
| Young-old (60 to 69 years) | 0.023 (-0.020, 0.067), 0.292 |
| Old-old (70 to 79 years) | -0.017 (-0.070, 0.035), 0.517 |
|  |  |
| **Poor health** |  |
|  |  |
| Children (6 to 19 years) | -0.0059 (-0.021, 0.009), 0.431 |
| Adults (20 to 59 years) | -0.0031 (-0.018, 0.012), 0.677 |
| Young-old (60 to 69 years) | -0.029 (-0.062, 0.005), 0.097 |
| Old-old (70 to 79 years) | -0.0043 (-0.056, 0.048), 0.870 |
|  |  |

Each model included an interaction between the rank variable for SII and year (categorical, included only 1992 and 2013) and adjusted for 5-year interval age, gender, marital status and 9-group prefecture. All analyses were adjusted for data clustering by household and weighted. Marital status was not included in children. 1992 is the reference, and the coefficients of an interaction term between the rank variable and year are reported in the table. The coefficients indicate the magnitude of change in SII in 2013 compared with 1992.

Sample size was 37,101for children, 113,533 for adults, 28,128 for young-old, and 18,392 for old-old.

Back to the contents list (p.1)

# FIGURES

## FIGURE A1 (A) Historical trends in the highest Nikkei Stock Average values on 1 January for Japan, and (B) GDP growth rates for Japan, United Kingdom and United States

Sources of data: (A) Nikkei Indexes. Historical Data in Nikkei 225. https://indexes.nikkei.co.jp/en/nkave/archives/data. (B) The World Bank. GDP growth (annual %). <https://data.worldbank.org/indicator>.

(A & B) Japan: red thick solid line, UK: light blue solid line, US: dark blue dashed line.

Back to the contents list (p.1)

## FIGURE A2 Inequality (adjusted Gini coefficient) in household income before and after tax, 1986-2013

****Adjusted for mean age of adults ≥20 years in household, age and gender of the household head, household size and prefecture (Wertz method).

Back to the contents list (p.1)

## FIGURE A3 Prevalence of age- and gender-standardized wellbeing and poor health by age-group, 1986-2013

Direct standardization method was used. The entire sample of analysis was used as the reference population.

Back to the contents list (p.1)

## FIGURE A4 Relative Index of Inequality in wellbeing based on household income after tax by age-group, 1992-2013

Dots represent RII for each year calculated separately for each survey year (see: supplementary appendix Table A4).

Line and confidence intervals (shadowed area) were calculated using coefficients and standard errors obtained from a quadratic time trend model. Reported p-values are for a quadratic trend term if quadratic trend term was statistically significant. Otherwise, p-values for a linear trend term from a linear trend model which does not include a quadratic term in the model was reported. (see: supplementary appendix Table A5).

All analyses were weighted using a survey weight and adjusted for categorical year, 5-year interval age, gender, marital status and 9-group prefecture, and robust standard errors were estimated using cluster sandwich estimator due to data clustering by household. In analysis of children, marital status was not included.

Back to the contents list (p.1)

## FIGURE A5 Relative Index of Inequality in poor health based on household income after tax for age-group, 1992-2013

Dots represent RII for each year calculated separately for each survey year (see: supplementary appendix Table A6).

Line and confidence intervals (shadowed area) were calculated using coefficients and standard errors obtained from a quadratic time trend model. Reported p-values are for a quadratic trend term if quadratic trend term was statistically significant. Otherwise, p-values for a linear trend term from a linear trend model which does not include a quadratic term in the model was reported. (see: supplementary appendix Table A7).

All analyses were weighted using a survey weight and adjusted for categorical year, 5-year interval age, gender, marital status and 9-group prefecture, and robust standard errors were estimated using cluster sandwich estimator due to data clustering by household. In analysis of children, marital status was not included.

Back to the contents list (p.1)

## FIGURE A6 Prevalence of age-standardized wellbeing and poor health by age-group and gender, 1986-2013

Age were held at mean levels of each age-group. Wellbeing: red, poor health: blue, solid line: male, dashed line: female.

Back to the contents list (p.1)

## FIGURE A7 Slope Index of Inequality in wellbeing based on household income after tax for age-group and gender, 1992-2013

Solid line with hollow circles: male, dashed line with triangles: female. Q: Quadratic time trend, L: Linear time trend.

Dots represent SII for each year calculated separately for each survey year (see: supplementary appendix Table A10).

Line and confidence intervals (shadowed area) were calculated using coefficients and standard errors obtained from a quadratic time trend model. Reported p-values are for a quadratic trend term if quadratic trend term was statistically significant. Otherwise, p-values for a linear trend term from a linear trend model which does not include a quadratic term in the model was reported. (see: supplementary appendix Table A11).

All analyses were weighted using a survey weight and adjusted for categorical year, 5-year interval age, marital status and 9-group prefecture, and robust standard errors were estimated using cluster sandwich estimator due to data clustering by household. n analysis of children, marital status was not included.

Back to the contents list (p.1)

## FIGURE A8 Slope Index of Inequality in poor health based on household income after tax for age-group and gender, 1992-2013

Solid line with hollow circles: male, dashed line with triangles: female. Q: Quadratic time trend, L: Linear time trend.

Dots represent SII for each year calculated separately for each survey year (see: supplementary appendix Table A13).

Line and confidence intervals (shadowed area) were calculated using coefficients and standard errors obtained from a quadratic time trend model. Reported p-values are for a quadratic trend term if quadratic trend term was statistically significant. Otherwise, p-values for a linear trend term from a linear trend model which does not include a quadratic term in the model was reported. (see: supplementary appendix Table A14).

All analyses were weighted using a survey weight and adjusted for categorical year, 5-year interval age, marital status and 9-group prefecture, and robust standard errors were estimated using cluster sandwich estimator due to data clustering by household. In analysis of children, marital status was not included.

Back to the contents list (p.1)

## FIGURE A9 Relative Index of Inequality in wellbeing based on household income after tax for age-group and gender, 1992-2013

Solid line with hollow circles: male, dashed line with triangles: female. Q: Quadratic time trend, L: Linear time trend.

Dots represent SII for each year calculated separately for each survey year (see: supplementary appendix Table A10).

Line and confidence intervals (shadowed area) were calculated using coefficients and standard errors obtained from a quadratic time trend model. Reported p-values are for a quadratic trend term if quadratic trend term was statistically significant. Otherwise, p-values for a linear trend term from a linear trend model which does not include a quadratic term in the model was reported. (see: supplementary appendix Table A11).

All analyses were weighted using a survey weight and adjusted for categorical year, 5-year interval age, marital status and 9-group prefecture, and robust standard errors were estimated using cluster sandwich estimator due to data clustering by household. In analysis of children, marital status was not included.

Back to the contents list (p.1)

## FIGURE A10 Relative Index of Inequality in poor health based on household income after tax for age-group and gender, 1992-2013

Solid line with hollow circles: male, dashed line with triangles: female. Q: Quadratic time trend, L: Linear time trend.

Dots represent SII for each year calculated separately for each survey year (see: supplementary appendix Table A13).

Line and confidence intervals (shadowed area) were calculated using coefficients and standard errors obtained from a quadratic time trend model. Reported p-values are for a quadratic trend term if quadratic trend term was statistically significant. Otherwise, p-values for a linear trend term from a linear trend model which does not include a quadratic term in the model was reported. (see: supplementary appendix Table A14).

All analyses were weighted using a survey weight and adjusted for categorical year, 5-year interval age, marital status and 9-group prefecture, and robust standard errors were estimated using cluster sandwich estimator due to data clustering by household. analysis of children, marital status was not included.

Back to the contents list (p.1)
